# Interfacial Electric Field Engineering of High‐Entropy Heterostructures Triggering Self‐Limiting Reconstruction for Industrial‐Scale Electrocatalysis

**DOI:** 10.1002/advs.77140

**Published:** 2026-08-21

**Authors:** Liang Yan, Yueqi Zhang, Bing Zhang, Jingyi Ye, Hao Li

**Affiliations:** ^1^ School of Chemistry and Materials Engineering Huizhou University Huizhou China; ^2^ School of Intelligent Manufacturing Huzhou College Huzhou China

**Keywords:** dynamic surface reconstruction, high‐entropy heterostructures, industrial‐scale electrocatalysis, interfacial electric field, oxygen evolution reaction

## Abstract

Severe structural degradation of electrocatalysts under ampere‐level operation remains a formidable bottleneck for industrial‐scale green hydrogen production. Herein, an interfacial electric field engineering strategy is reported by integrating a high‐entropy tungstate (FeCoNiMnWO_4_, HEW) with CeO_2_ nanoparticles. Theoretical calculations and direct surface potential mapping reveal that the intrinsic Fermi level offset drives spontaneous electron transfer, establishing a robust built‐in electric field (BIEF) at the heterointerface. In situ spectroscopy and comprehensive thermodynamic analyses uncover that this engineered field acts as a critical regulator, inducing a “self‐limiting” surface amorphization. The BIEF thermodynamically triggers the sacrificial leaching of metastable W species to form a highly active metal oxyhydroxide layer, while a high barrier for continuous lattice oxygen consumption prevents catastrophic bulk degradation. This regulated phase evolution directs the steady‐state catalysis exclusively via the adsorbate evolution mechanism (AEM), lowering the thermodynamic barrier of the rate‐determining step (^*^OH → ^*^O). Consequently, the HEW‐CeO_2_ heterostructure delivers exceptional oxygen evolution reaction (OER) performance, requiring only 337 mV overpotential to sustain 1000 mA cm^−2^ for over 100 h. This work establishes a versatile paradigm for coupling interfacial field engineering with dynamic phase evolution in advanced energy conversion.

## Graphical abstract

An interfacial electric field engineering strategy is proposed to trigger the self‐limiting surface reconstruction of high‐entropy heterostructures. Driven by this built‐in field, the controlled sacrificial leaching of W evolves a robust active layer, enabling exceptional oxygen evolution performance and ultrastability at ampere‐level current densities for industrial‐scale green hydrogen production.

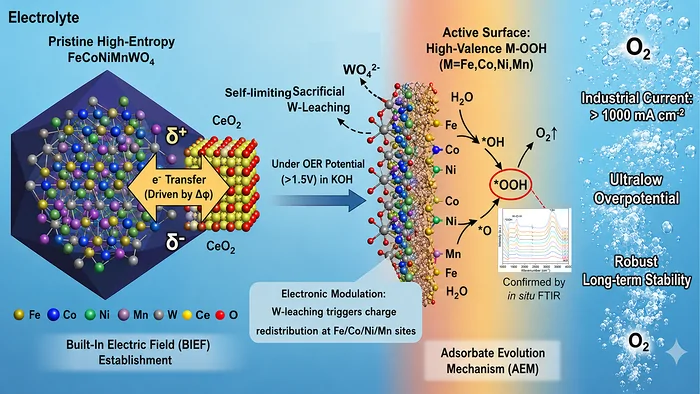

## 1 Introduction

The escalating global energy crisis and intensifying climate change, primarily driven by excessive reliance on fossil fuels, underscore the urgent need for a transition toward clean and sustainable energy systems [1]. Green hydrogen (H_2_), produced via water electrolysis powered by renewable sources, is widely regarded as a cornerstone of the future carbon‐neutral economy [2]. However, the overall efficiency of water electrolysis is severely constrained by the anodic oxygen evolution reaction (OER), a complex four‐electron–proton‐coupled process (2H_2_O → O_2_ + 4H^+^ + 4𝑒^−^) plagued by sluggish kinetics [3]. This kinetic bottleneck necessitates large overpotentials, resulting in substantial energy losses. Although state‐of‐the‐art noble‐metal catalysts such as IrO_2_ and RuO_2_ exhibit excellent OER activity, their high cost, scarcity, and poor durability in alkaline media significantly hinder their large‐scale application [4]. Consequently, there is a pressing demand for earth‐abundant, highly active, and robust electrocatalysts [5]. Considerable progress has been made with transition metal‐based materials, including (oxy)hydroxides [6], phosphides [7], and nitrides [8], among others [9]. Nevertheless, a critical performance gap remains: most reported catalysts are evaluated at a benchmark current density of 10 mA cm^−2^, far below the industrially relevant levels (e.g., 500–1000 mA cm^−2^) required for practical electrolyzers [10]. Under such ampere‐level conditions, conventional catalysts often suffer from severe performance degradation, structural instability, and mass transport limitations (e.g., gas bubble accumulation), rendering them unsuitable for long‐term operation [11]. Therefore, the rational design of catalysts that maintain high activity and durability under industrial‐level current densities remains a formidable challenge.

High‐entropy materials (HEMs) have recently emerged as a promising class of electrocatalysts to overcome this limitation. The “cocktail effect” arising from multiple metal constituents can stabilize active phases and disrupt linear scaling relationships of intermediate adsorption energies, thereby enhancing catalytic performance [12]. In particular, multi‐metal tungstates (MWO_4_) have attracted growing attention. Rather than serving merely as stable oxides, these materials act as ideal precatalysts capable of undergoing deep surface reconstruction to generate defect‐rich, catalytically active oxyhydroxide layers—a transformation often overlooked but critical for boosting activity. However, the intrinsic activity of single‐phase tungstates remains limited by sluggish charge transfer and suboptimal electronic configurations [13].

Interfacial engineering via heterostructure construction offers a powerful strategy to address these limitations. Among various candidates, CeO_2_ stands out due to its high oxygen storage capacity and large work function (Φ), which enables it to act as an efficient electron acceptor that modulates the electronic environment of adjacent transition metals. This role is further supported by our DFT‐derived Φ analysis. More importantly, the formation of a heterojunction between MWO_4_ and CeO_2_ induces a BIEF arising from the Fermi level difference. This internal field serves as a potent driving force to (i) accelerate interfacial charge transfer, (ii) enhance the electrophilicity of active centers, and (iii) precisely tune the adsorption free energies (Δ𝐺) of key intermediates, thereby lowering the reaction barrier [14]. Harnessing such BIEF effects provides an elegant route to couple high‐entropy design with field‐induced electronic modulation.

Herein, we propose an interfacial electric field engineering strategy to address the structural stability challenge of electrocatalysts under industrial‐level current densities. By rationally integrating a high‐entropy tungstate (HEW) with CeO_2_ nanoparticles, a strong directional built‐in electric field (BIEF) is established at the heterointerface, driven by the intrinsic work function offset. Within this precisely designed high‐entropy matrix, the constituent metals transcend mere structural scaffolds to synergistically drive electrocatalysis. Specifically, while W undergoes controlled sacrificial leaching, Ni and Co serve as the intrinsic active centers capable of forming highly active oxyhydroxides (NiOOH/CoOOH). Concurrently, Mn acts as a vital electronic modulator to optimize the local coordination environment, supported by Fe as the primary electron donor that profoundly enriches the electron density of the Co active sites. This engineered BIEF does not merely serve as an electronic modulator but acts as a dynamic trigger for regulated structural evolution. In situ spectroscopic evidence reveals that the BIEF‐modulated extraction of metastable W species enables the seamless transformation of the pre‐catalyst into a robust, defect‐rich metal oxyhydroxide active layer without causing bulk structural collapse. This field‐induced phase evolution enables the heterostructure to deliver exceptional OER performance, sustaining a record‐low overpotential of 337 mV at an industrial‐scale current density of 1000 mA cm^−2^ with over 100 h of stability. This work provides a new design paradigm for developing resilient high‐entropy functional materials via precise interfacial field engineering.

## 2 Results and Discussion

### 2.1 Synthesis and Structural Characterizations

**FIGURE 1:**
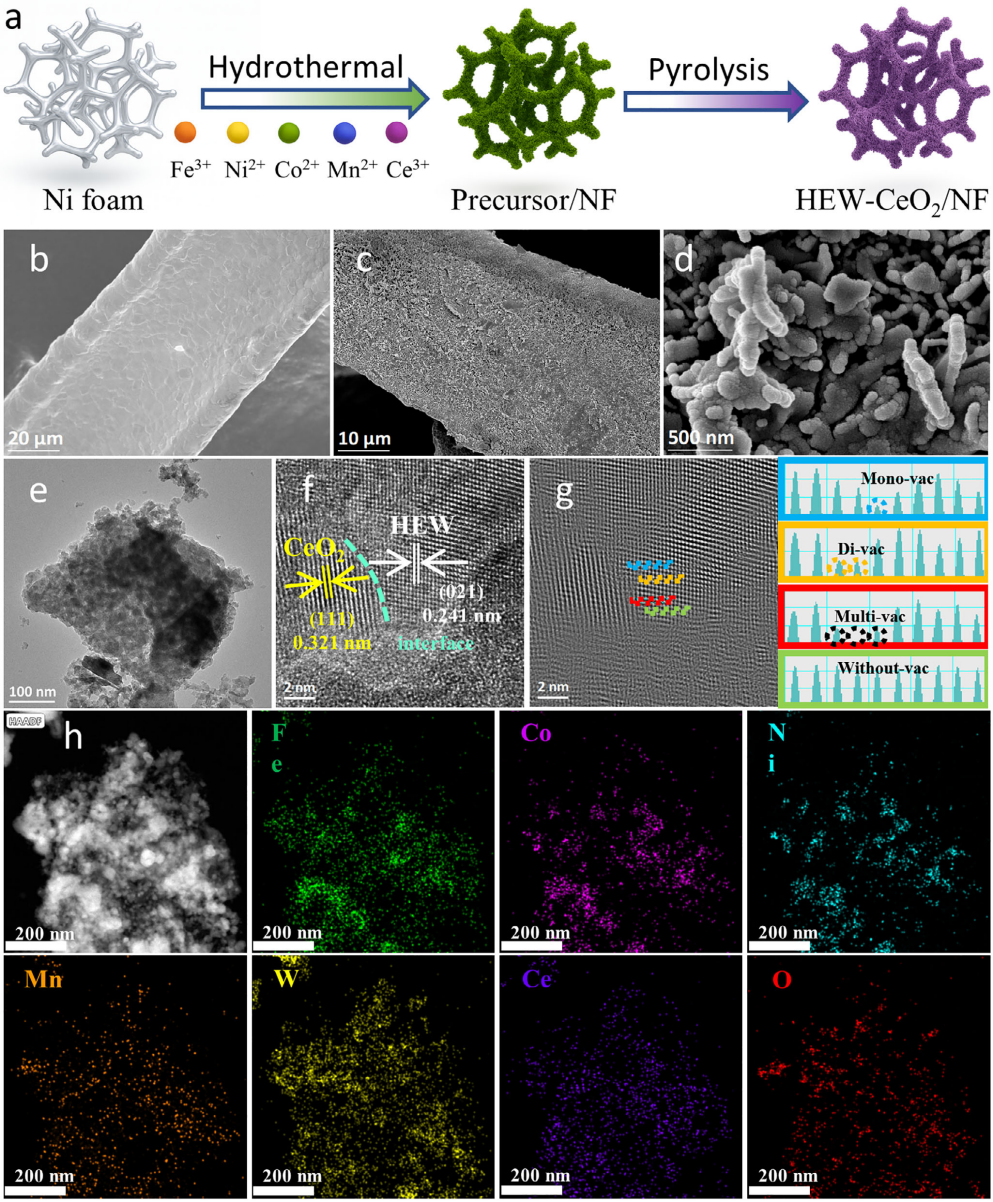
Synthesis and morphological characterization of HEW‐CeO_2_/NF. (a) Schematic illustration of the synthesis process for HEW‐CeO_2_/NF. SEM images of (b) bare NF and (c,d) HEW‐CeO_2_/NF at different magnifications. (e) TEM, (f) HRTEM, and (g) the corresponding Inverse‐FFT image of (f). The line scan profiles for (g) are provided on the right. The blue, yellow, red, and green lines in (g) denote mono‐vacancies, di‐vacancies, multi‐vacancies, and vacancy‐free regions, respectively. (h) STEM‐EDX elemental mapping images of HEW‐CeO_2_.

The rational design of the hierarchical HEW‐CeO_2_ heterostructure supported on conductive nickel foam (NF) was achieved through a facile two‐step strategy, as schematically illustrated in Figure 1a. The process involved a one‐step hydrothermal reaction to grow the precursor arrays onto the 3D NF substrate, followed by a controlled pyrolysis treatment in an inert atmosphere to induce crystallization and establish the final heterojunction. For comparison, the pristine HEW and CoNiWO_4_ control samples were also synthesized (Figure S1). The morphology of the as‐prepared catalyst was first investigated using scanning electron microscopy (SEM). Figure 1b displays the smooth, scaffold‐like structure of the bare NF substrate. After the growth process, the NF is uniformly and densely covered with a 3D, hierarchically porous catalyst layer (Figure 1c). This open 3D architecture is intrinsic to high‐performance electrodes, as it significantly amplifies the electrochemical active surface area (ECSA) and facilitates mass transport by allowing deep electrolyte infiltration and rapid O_2_ bubble release. The high‐magnification SEM image (Figure 1d) reveals a hierarchically porous surface composed of interconnected flake‐like nanostructures. The coexistence of these anisotropic features forms a rugged, coral‐like architecture that promotes abundant exposure of active sites and facilitates mass transport, contributing to enhanced electrocatalytic performance. The microstructure and interfacial properties of the HEW‐CeO_2_ catalyst were further elucidated by transmission electron microscopy (TEM). The low‐magnification TEM image (Figure 1e) reveals that the catalyst consists mainly of flake‐like nanosheets assembled into a porous network, in agreement with the morphology observed in SEM. High‐resolution TEM (HRTEM) was employed to provide definitive evidence of the heterostructure formation at the atomic level (Figure 1f). Two distinct sets of lattice fringes are clearly resolved, indicating intimate interfacial coupling. The lattice spacing measured at 0.321 nm corresponds perfectly to the (111) crystal plane of cubic CeO_2_ [15]. Adjacent to this, the lattice fringe of 0.241 nm is unambiguously assigned to the (021) plane of the high‐entropy tungstate phase. This observation unequivocally confirms the successful construction of an atomic‐scale HEW‐CeO_2_ heterojunction, which serves as the structural prerequisite for generating the BIEF and inducing interfacial electronic modulation. To further analyze the atomic‐scale features, the corresponding inverse Fast Fourier Transform (IFFT) image was generated from the HRTEM data (Figure 1g). The IFFT image and its corresponding line scan profiles (right panel) reveal severe lattice distortions, a hallmark of high‐entropy materials. As color‐coded in the profiles, abundant mono‐vacancies (blue), di‐vacancies (yellow), and multi‐vacancies (red) are prevalent throughout the lattice, contrasting with the ordered, vacancy‐free regions (green) [16]. This highly distorted structure, stemming from the “cocktail effect” of multi‐element mixing, creates a rich density of surface defects (e.g., oxygen vacancies) at the interface. These defects are expected to act as powerful electronic modulators, optimizing the adsorption energy of OER intermediates and accelerating charge transfer kinetics. Finally, scanning TEM (STEM) coupled with energy‐dispersive X‐ray (EDX) elemental mapping was performed to verify the composition (Figure 1h). The images confirm the homogeneous distribution of all constituent elements: Fe, Co, Ni, Mn, W, Ce, and O. Crucially, the intimate co‐location of all metallic elements provides strong evidence for the formation of a uniform high‐entropy heterostructure at the nanoscale. This ensures a maximized density of interfacial active sites where the BIEF‐driven synergistic catalysis can occur.

**FIGURE 2:**
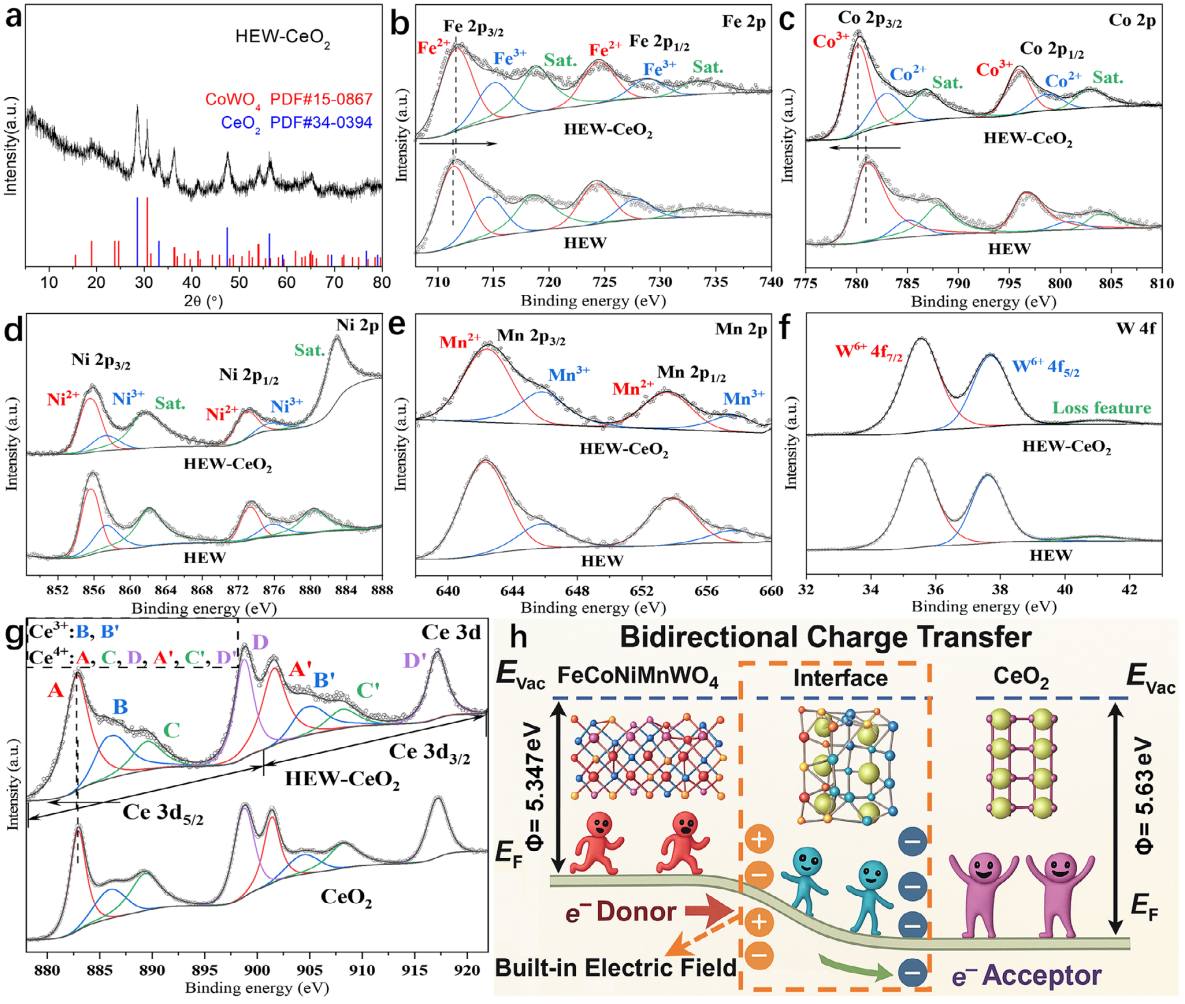
XRD and XPS characterizations. (a) XRD pattern of HEW‐CeO_2_/NF. High‐resolution XPS spectra of (b) Fe 2p, (c) Co 2p, (d) Ni 2p, (e) Mn 2p, (f) W 4f, (g) Ce 3d. (h) The schematic illustrates the Mn^−^ dominated multidirectional charge redistribution. Mn acts as the electron donor (δ^+^), transferring electrons simultaneously to the internal Co/Ni sites (δ^−^) and interfacial Ce sites (δ^−^), driven by the BIEF.

To determine the crystal structure and phase composition, X‐ray diffraction (XRD) was performed. As shown in Figure 2a, the XRD pattern of the HEW‐CeO_2_ heterostructure displays two distinct sets of diffraction peaks. The peaks indexed in blue match well with the cubic fluorite CeO_2_ (JCPDS No. 34–0394), while those marked in red are in perfect agreement with the monoclinic wolframite structure (isostructural with CoWO_4_, JCPDS No. 15–0867), confirming the successful crystallization of the high‐entropy HEW phase. The sharp diffraction peaks with no observable impurities validate the successful construction of the high‐purity heterostructure. X‐ray photoelectron spectroscopy (XPS) was employed to probe the surface chemical states and, crucially, the interfacial electronic interactions. The survey scan of XPS spectra (Figure S2) reveals the presence of Fe, Co, Ni, Mn, W, Ce, and O elements in the HEW‐CeO_2_ heterostructure, which agrees with the above elemental mappings. Figure 2b–e displays the high‐resolution spectra of Fe 2p (Figure 2b), Co 2p (Figure 2c), Ni 2p (Figure 2d), and Mn 2p (Figure 2e) for the HEW‐CeO_2_ hybrid. All four transition metals exhibit characteristic spin–orbit doublets accompanied by satellite peaks, confirming the coexistence of mixed‐valence states (M^2+^/M^3+^). This rich multivalent environment is pivotal for facilitating reversible redox cycling during the OER process [17, 18, 19]. The W 4f spectrum (Figure 2f) displays a characteristic doublet at 35.50/37.69 eV, identifying tungsten primarily in the W^6+^ oxidation state [20]. Meanwhile, the Ce 3d spectrum (Figure 2g) reveals a mixture of Ce^3+^ and Ce^4+^ species [21]. Notably, the presence of Ce^3+^ is intrinsically linked to the formation of oxygen vacancies (O_v_) to maintain charge neutrality. The impact of heterointerface engineering on defect generation was quantified by the O 1s spectra (Figure S3a). The peaks are resolved into lattice oxygen (O_L_), oxygen vacancies (O_v_), and adsorbed hydroxyls (O_ads_) [22]. The relative concentration of vacancies (O_v_/O_L_), calculated from integrated areas (Table S1) [23], increases significantly from 0.74 in pure HEW to 1.20 in HEW‐CeO_2_. This finding confirms that CeO_2_ introduction promotes abundant O_v_ generation [24], which serves to optimize electrical conductivity and intermediate binding [25]. This conclusion is further substantiated by Electron Paramagnetic Resonance (EPR) spectroscopy (Figure S3b). Both samples exhibit a symmetrical signal at g = 2.003, a signature of unpaired electrons trapped at O_v_ sites [26]. Critically, the markedly stronger signal intensity for the heterostructure provides direct spectroscopic evidence of a higher defect concentration, aligning perfectly with the XPS results.

To gain deeper insights into the electronic coupling at the heterostructure interface, a comprehensive spectroscopic and theoretical analysis was performed. Crucially, a comparative XPS analysis unveils a BIEF‐driven multidirectional charge redistribution mechanism (Figure 2h). Relative to the single‐phase HEW, the Fe 2p binding energy in the HEW‐CeO_2_ heterostructure exhibits a distinct positive shift (+0.26 eV), identifying Fe as the primary electron donor within the high‐entropy matrix (Table S2). These released electrons are delocalized and accepted by neighboring catalytic sites, predominantly Co (exhibiting a massive negative shift of −0.79 eV) within the tungstate lattice, as well as interfacial Ce (−0.10 eV) in the CeO_2_ domain [27]. In contrast, the Ni 2p, Mn 2p, and W 4f spectra show relatively minor shifts (< 0.1 eV), suggesting that Ni and Mn serve as vital structural and electronic modulators to stabilize the multi‐principal‐element matrix, while the metastable W species anchor the local electronic environment prior to their sacrificial leaching during reconstruction. This experimental evidence correlates perfectly with the band alignment model (Figure S4), where the lower Φ of HEW (5.347 eV) relative to CeO_2_ (5.63 eV) drives spontaneous electron diffusion upon contact to equilibrate the Fermi levels (EF). As illustrated in the schematic, this charge migration establishes a robust BIEF directed from the tungstate toward the oxide, inducing upward band bending on the tungstate side. The resulting electron depletion at Fe sites (δ^+^) and profound accumulation at acceptor sites (Co and Ce, δ^−^) create a highly polarized surface that enhances the electrophilicity of the active centers. To provide direct spatial visualization of this built‐in electric field, Kelvin probe force microscopy (KPFM) was employed (Figure S5). The KPFM surface potential profile reveals a distinct and continuous potential step of approximately 148 mV across the HEW and CeO_2_ adjacent domains. This experimentally observed macroscopic electrostatic contrast perfectly corroborates the DFT‐derived work‐function offset, providing unambiguous physical proof for the spontaneous, long‐range interfacial charge transfer and the successful establishment of the BIEF. As confirmed by subsequent in situ characterization, this BIEF‐induced pre‐oxidation effectively lowers the energy barrier for the rate‐determining step (^*^OH → ^*^O) and significantly accelerates OER kinetics, ultimately enabling stable ampere‐level water oxidation.

**FIGURE 3:**
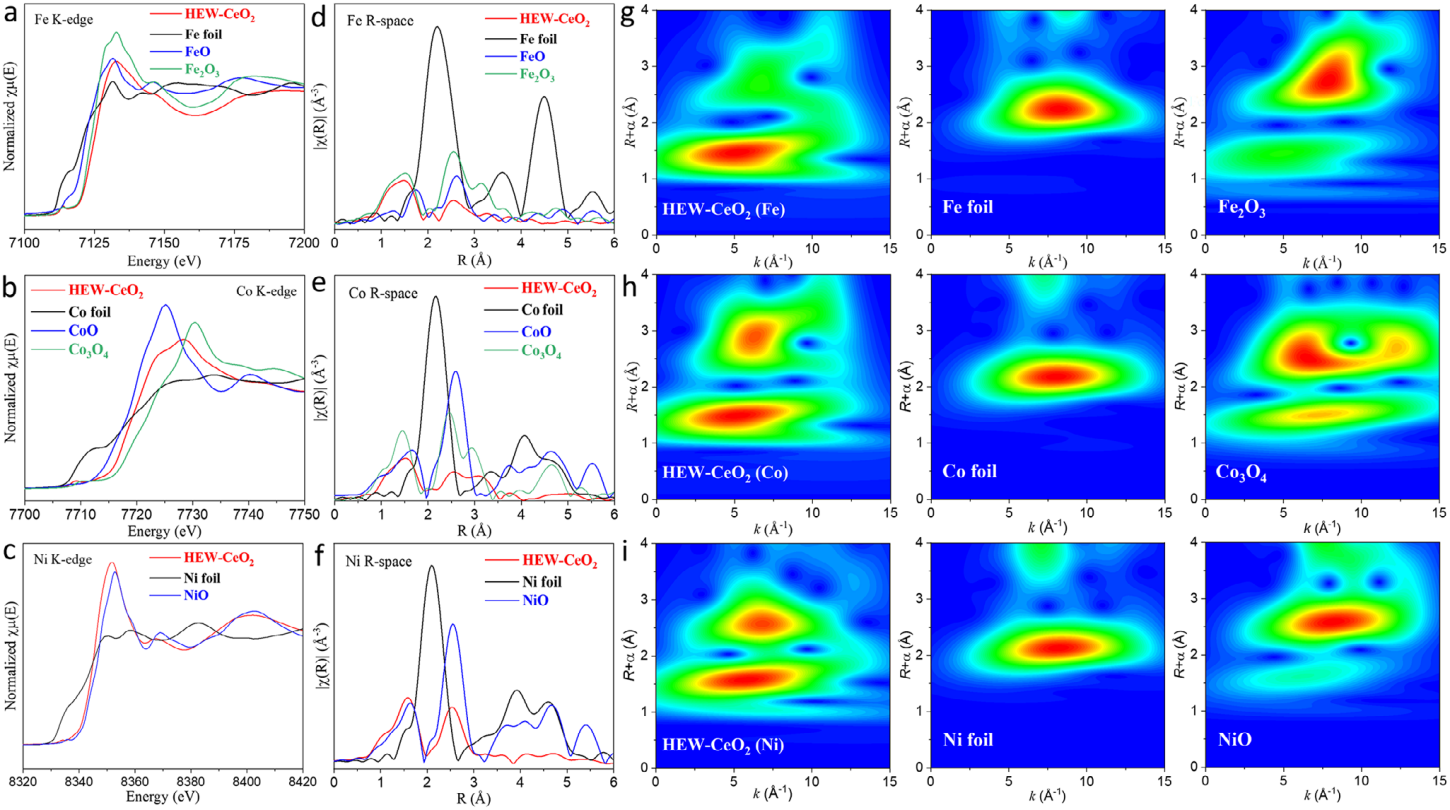
XAFS characterizations. (a) Fe K‐edge XANES and (d) corresponding FT‐EXAFS spectra. (b) Co K‐edge XANES and (e) corresponding FT‐EXAFS spectra. (c) Ni K‐edge XANES and (f) corresponding FT‐EXAFS spectra. (g) Fe K‐edge WTs of HEW‐CeO_2_, Fe foil, and Fe_2_O_3_. (h) Co K‐edge WTs of HEW‐CeO_2_, Co foil, and Co_3_O_4_. (i) Ni K‐edge WTs of HEW‐CeO_2_, Ni foil, and NiO.

To elucidate the atomic‐scale structure and electronic configuration of the HEW‐CeO_2_/NF heterostructure, comprehensive X‐ray absorption fine structure (XAFS) analyses were conducted. Normalized X‐ray absorption near‐edge structure (XANES) spectra were first analyzed to unravel the valence evolution of the transition metal species. As shown in Figure 3a,b, the Fe and Co K‐edge absorption thresholds exhibit a distinct positive shift relative to their respective metallic foils, positioning them between standard divalent (M^2+^) and trivalent (M^3+^) references. This explicitly indicates the presence of mixed‐valence configurations (Fe^2+^/Fe^3+^ and Co^2+^/Co^3+^). In contrast, the Ni K‐edge (Figure 3c) aligns closely with the NiO reference, confirming the predominance of the Ni^2+^ state. Crucially, these modulated electronic states are not merely intrinsic but are further optimized by the synergistic electron transfer between the high‐entropy tungstate matrix and the oxygen‐deficient CeO_2_. Rather than simple oxidation, this configuration represents an optimized electrophilicity of the active centers. Such a specific electronic landscape is expected to balance the adsorption energetics of key oxygenated intermediates (e.g., ^*^OH, ^*^O, ^*^OOH), thereby facilitating the reversible redox kinetics required for the OER. Fourier‐transformed extended X‐ray absorption fine structure (FT‐EXAFS) spectra provide fingerprint information on the local coordination environment and lattice connectivity. As depicted in Figure 3d–f, all three transition metals (Fe, Co, Ni) exhibit a prominent peak at approximately 1.5 Å, corresponding to the first coordination shell of metal–oxygen (M─O) bonds. Notably, a second coordination shell emerges in the 2.5–3.0 Å range, attributable to metal–metal (M─M) or metal‐tungsten (M─W) scattering within the crystalline wolframite framework. The absence of any peak characteristic of metallic bonding (M─M at ∼2.2 Å) confirms that the transition metals are atomically incorporated into the specific lattice sites of the high‐entropy structure, forming a homogeneous solid solution rather than aggregating into amorphous clusters or discrete oxide phases. To further disentangle the backscattering signals in the second coordination shell, wavelet transform (WT) analysis was employed (Figure 3g–i), offering simultaneous resolution in both *k*‐ and *R*‐space. The WT contour plots of HEW‐CeO_2_ reveal two distinct intensity maxima: a low ‐*k* signal (*k* ≈ 4–6 Å^−1^) associated with light oxygen atoms (M─O scattering), and a prominent higher ‐*k* signal (spanning *k* ≈ 6–11 Å^−1^) corresponding to heavier M─M/M─W coordination. Notably, compared to the relatively localized M─M signals in the standard simple oxides (Fe_2_O_3_, Co_3_O_4_, NiO), the second‐shell WT signals of HEW‐CeO_2_ exhibit broader *k*‐space distributions. This distinctive broadening further validates the atomic‐level incorporation of transition metals into the complex high‐entropy tungstate lattice with heavy W scattering. Concurrently, the complete absence of foil‐like low‐*R*/high‐*k* intensity spots unambiguously excludes the presence of metallic segregation or isolated simple oxide impurity phases. This confirms the structural integrity of the high‐entropy lattice, which serves as the robust foundation for the subsequent BIEF‐driven surface reconstruction.

### 2.2 OER Performance

**FIGURE 4:**
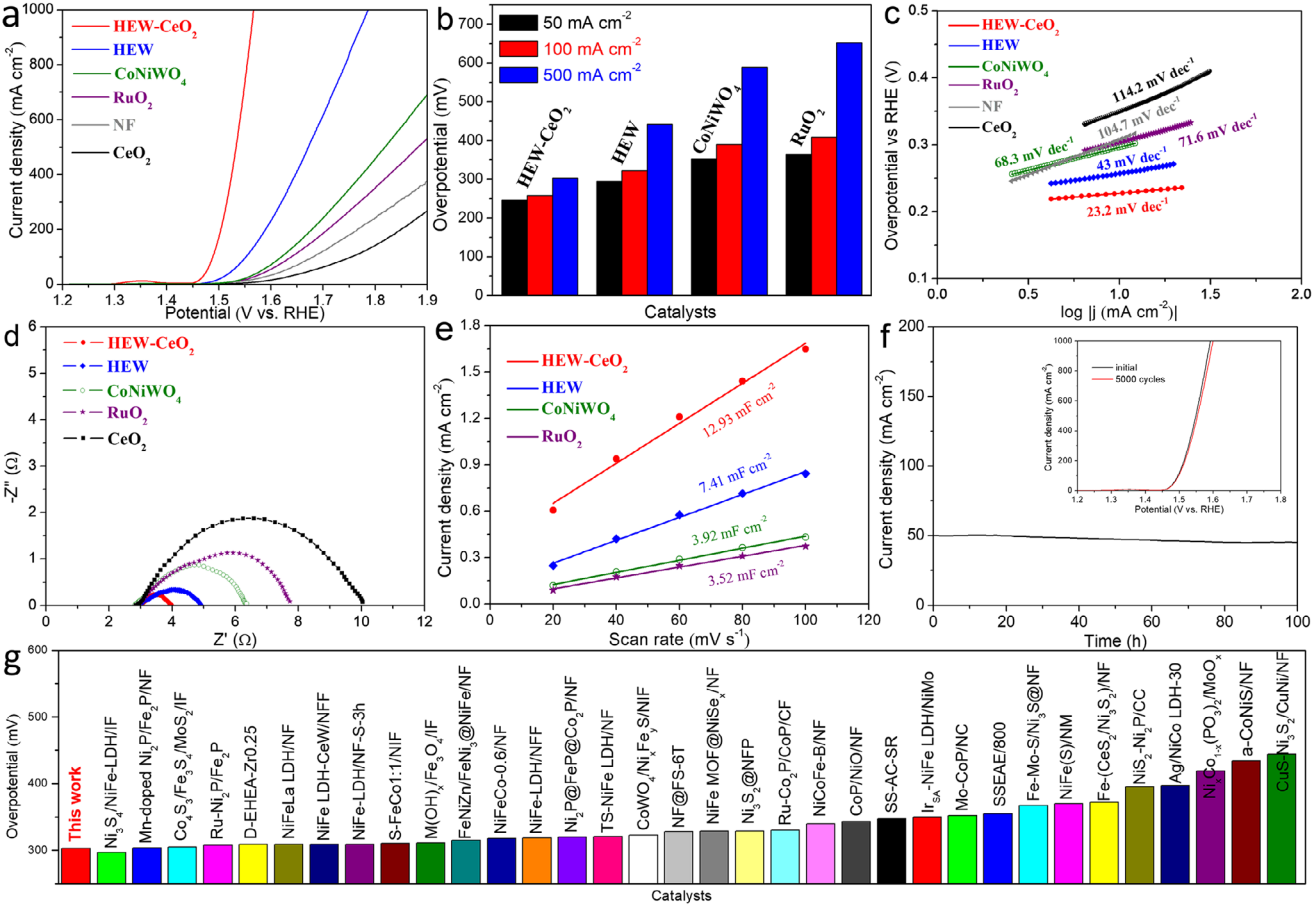
Electrochemical OER performance. (a) LSV curves, (b) Overpotentials at current densities of 50, 100, and 500 mA cm^−2^, (c) Tafel slopes, (d) EIS, (e) C_dl_ values, and (f) i–t curves under constant potential for OER stability evaluation (inset: LSV curves before and after 5000 cycles). (g) Comparison of HEW‐CeO_2_/NF with reported electrocatalysts at 500 mA cm^−2^.

The electrocatalytic OER activity was assessed in 1.0 m KOH using a standard three‐electrode configuration. Figure 4a presents the iR‐compensated LSV profiles of the high‐entropy HEW‐CeO_2_/NF heterostructure, benchmarked against single‐phase HEW/NF, CoNiWO_4_/NF, CeO_2_/NF, commercial RuO_2_/NF, and bare NF. Among all investigated samples, HEW‐CeO_2_/NF delivers the superior catalytic performance. It requires remarkably low overpotentials of only 247, 258, and 303 mV to drive current densities of 50, 100, and 500 mA cm^−2^ (Figure 4b), respectively. These metrics represent a substantial improvement over the comparative references, including HEW/NF (296, 322, and 442 mV) and the commercial RuO_2_/NF benchmark (365, 409, and 652 mV), underscoring the efficacy of the BIEF‐driven heterostructure strategy. To further rule out mere compositional effects, a physical mixture of HEW and CeO_2_ (HEW + CeO_2_) was also evaluated under identical conditions (Figure S6). The significantly inferior performance of the physical mixture compared to the engineered heterostructure unambiguously verifies that atomic‐level interfacial coupling and the resulting BIEF are indispensable for unlocking the exceptional OER activity, rather than the simple coexistence of the two components. The reaction kinetics were further deciphered by Tafel analysis (Figure 4c). The HEW‐CeO_2_/NF catalyst exhibits an exceptionally low Tafel slope of 23.2 mV dec^−1^, significantly outperforming RuO_2_/NF (71.6 mV dec^−1^) and other control samples (43−114.2 mV dec^−1^). This rapid kinetic response indicates that the rate‐determining step (RDS) is effectively accelerated, attributed to the optimized adsorption energetics of ^*^OH/^*^O intermediates modulated by the BIEF. Consistent with this, Electrochemical Impedance Spectroscopy (EIS) reveals that the heterostructure possesses the lowest charge transfer resistance (R_ct_), confirming the fastest interfacial electron transfer during the OER process (Figure 4d). To quantify the intrinsic active site density, the electrochemical double‐layer capacitance (C_dl_) was determined (Figure S7). As summarized in Figure 4e, HEW‐CeO_2_/NF achieves the highest C_dl_ of 12.93 mF cm^−2^, surpassing the single‐phase tungstate (7.41 mF cm^−2^) and RuO_2_/NF (3.52 mF cm^−2^). This enhanced electrochemically active surface area (ECSA) stems from the synergistic combination of the hierarchical porous architecture and the severe lattice distortions intrinsic to the high‐entropy lattice, which maximize the exposure of active sites. The operational durability, a critical criterion for industrial application, was rigorously evaluated. Continuous cycling for 5000 CV cycles resulted in negligible polarization shifts (Inset of Figure 4f). Furthermore, long‐term chronoamperometry demonstrated robust stability for over 100 h at high current densities without apparent decay, confirming the structural resilience of the electrode. Notably, the overall performance compares favorably against state‐of‐the‐art non‐noble metal electrocatalysts recently reported in the literature (Figure 4g, Table S3). Beyond the exceptional catalytic performance, the practical scalability and cost‐effectiveness of the HEW‐CeO_2_/NF heterostructure further underscore its immense potential for industrial application. From a compositional perspective, the catalyst is exclusively constructed from earth‐abundant transition metals (Fe, Co, Ni, Mn, W, and Ce), completely circumventing the reliance on scarce and cost‐prohibitive noble metals like Ir and Ru. Furthermore, the synthesis relies on a straightforward, template‐free hydrothermal growth followed by pyrolysis directly on a commercially available, low‐cost nickel foam substrate. This binder‐free, self‐supported configuration not only ensures robust mechanical adhesion for long‐term chronoamperometric stability but is also inherently compatible with existing large‐area roll‐to‐roll manufacturing processes, laying a solid foundation for its deployment in practical, large‐scale water electrolyzers.

To unravel the origin of this sustained activity, post‐OER characterization was conducted to probe the active phase evolution. While SEM and TEM micrographs confirm that the macroscopic hierarchical architecture of the catalyst remains largely intact (Figure S8a,b), HRTEM analysis uncovers a profound surface reconstruction at the atomic level. The sustained activity and structural integrity despite W leaching suggest a strong interfacial pinning effect driven by the BIEF, which effectively anchors the reconstructed oxyhydroxide layer to the CeO_2_ support. This synergy prevents ‘over‐leaching’ of the bulk phase and structural pulverization, ensuring the catalyst maintains its robust performance even under the intense gas evolution at ampere‐level current densities. The surface reconstruction leads to the formation of poly‐metallic oxyhydroxides, where the resolved lattice fringes match well with the (310), (101), and (107) planes of the corresponding metal oxyhydroxides (Figure S8c). This formation of active oxyhydroxide species is further corroborated by post‐OER XPS analysis, which reveals a dynamic evolution in the chemical states of the surface metal centers. Specifically, the post‐OER spectra show significantly elevated ratios of Fe^3+^/Fe^2+^, Co^3+^/Co^2+^, and Mn^3+^/Mn^2+^ (Figure S9), indicating the in situ generation of high‐valence active species. Most strikingly, the post‐OER Ni 2p spectrum reveals the complete disappearance of Ni^2+^ features (Figure S10), with all detectable Ni species transforming into the trivalent state (Ni^3+^). Intriguingly, despite this oxidation, the binding energy shifts toward lower energy. This counter‐intuitive phenomenon, consistent with our in situ Raman observations of W leaching, is attributed to the “ligand release effect”. The removal of the strong electron‐withdrawing W^6+^ species from the lattice relieves the electron‐pulling effect on the Ni centers, causing a negative shift in binding energy even as the oxidation state increases. This confirms the in situ transformation of the pristine tungstate into a highly active, defect‐rich Fe‐Co‐Ni‐Mn oxyhydroxide layer, which serves as the true catalytic phase for ampere‐level water oxidation. Within this multi‐metallic active layer, the constituent transition metals play distinct yet highly synergistic roles that extend far beyond acting as mere structural components. Specifically, Ni and Co atoms serve as the primary catalytic engines, readily evolving into highly conductive and reactive oxyhydroxide complexes (NiOOH/CoOOH) under anodic potentials to provide abundant active sites. Concurrently, the incorporation of Fe acts as a powerful electron donor that profoundly enriches the local electron density of the adjacent Co sites. Working in tandem, Mn serves as a vital electronic regulator to optimize the overall d‐band centers and adsorption energetics of the highly distorted matrix. This interfacial electronic modulation effectively tailors the adsorption free energies of key oxygenated intermediates (^*^OH, ^*^O, and ^*^OOH), thereby lowering the thermodynamic barriers of the rate‐determining steps and breaking the conventional linear scaling relationships. Furthermore, from a structural perspective, the high‐entropy configuration involving Fe, Co, and Ni creates a severely distorted lattice matrix. This high configurational entropy provides significant thermodynamic resilience that effectively suppresses severe phase segregation and structural pulverization typically observed in conventional single‐ or binary‐metal oxides, thereby maintaining exceptional structural integrity and catalytic durability under industrial‐scale current densities.

### 2.3 Mechanism Analysis

**FIGURE 5:**
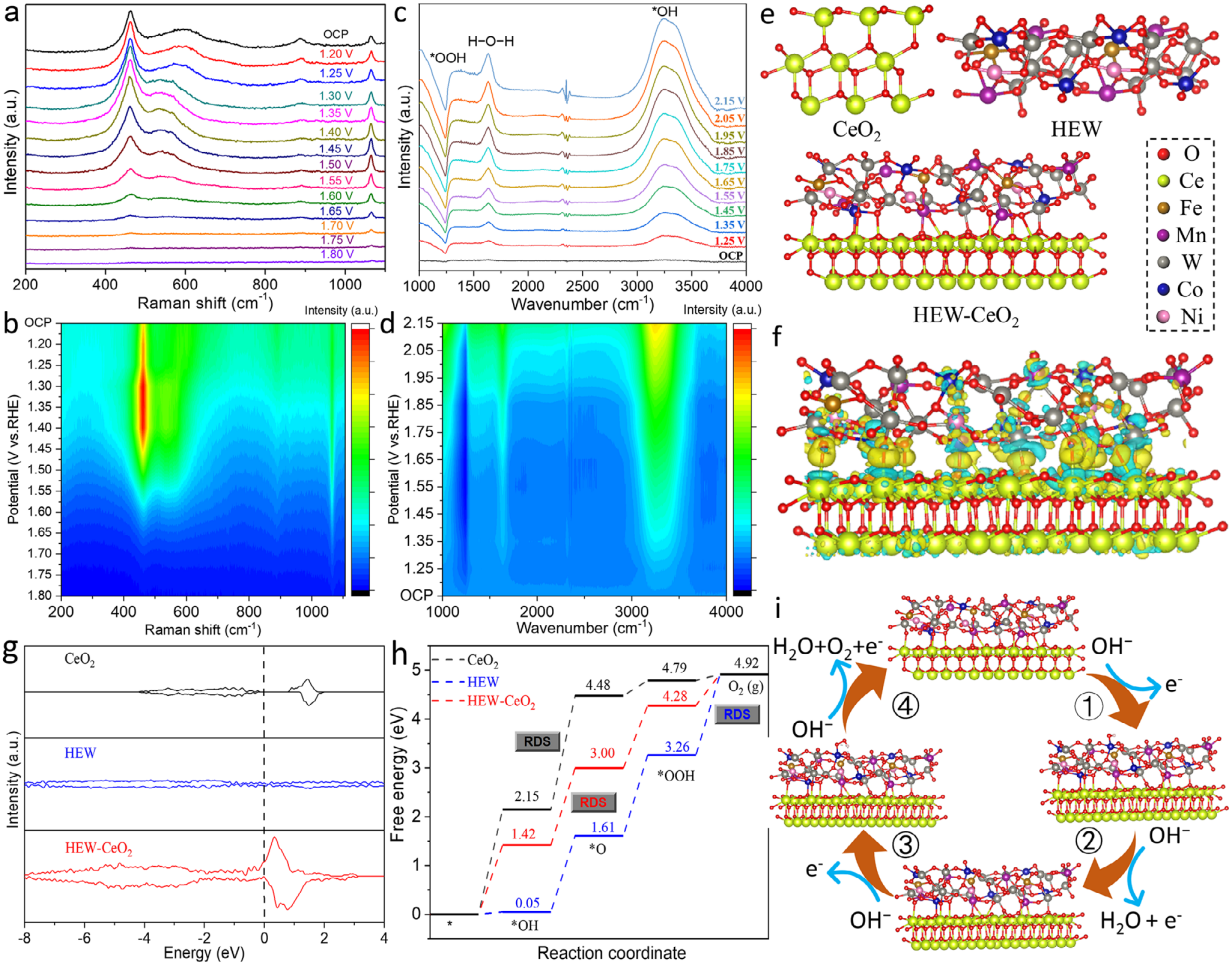
Mechanism explanation. (a) In situ Raman spectra and (b) corresponding contour plot; (c) in situ FTIRs spectra and (d) corresponding contour plot of HEW‐CeO_2_ during electrochemical OER at different potentials. (e) Optimized theoretical models of CeO_2_, HEW, and HEW‐CeO_2_ heterostructure. (f) Charge density difference map at the HEW‐CeO_2_ interface (cyan means electron accumulation and yellow means electron depletion). (g) DOS for CeO_2_, HEW, and HEW‐CeO_2_. (h) Adsorption energy of oxygen intermediates on CeO_2_, HEW, and HEW‐CeO_2_. (i) Schematic illustration of the proposed adsorbate evolution mechanism for OER.

To elucidate the real‐time structural evolution and the origin of the enhanced catalytic activity, in situ Raman spectroscopy coupled with ex situ XPS analysis was conducted on the HEW‐CeO_2_ heterostructure. As illustrated in Figure 5a, the pristine sample exhibits distinct Raman features at ∼880 and ∼460 cm^−^^1^, corresponding to the symmetric stretching of W─O bonds in the tungstate framework and the M─O/Ce─O lattice vibrations [28, 29], respectively. Upon applying an anodic potential (from OCP to 1.8 V), a dramatic spectral evolution is observed. The characteristic W─O and lattice peaks undergo a gradual attenuation and virtually vanish beyond 1.45 V, concomitant with the emergence of broad, diffuse bands in the low‐wavenumber region. This spectroscopic evidence strongly points to a potential‐driven surface reconstruction, involving the severe leaching of soluble tungstate species (W^6+^) and the transformation of the crystalline pre‐catalyst into an amorphous, catalytically active metal oxyhydroxide (M‐OOH) layer [30]. This leaching behavior represents a deliberate phase transformation rather than continuous degradation. As W ions are selectively removed from the surface, the resulting coordination vacancies are rapidly occupied by OH^−^ ions—a ‘vacancy‐filling’ mechanism that stabilizes the newly formed FeCoNiMn‐OOH active layer. Crucially, the extraordinary structural stability of this reconstructed oxyhydroxide layer under ampere‐level operation is ensured by a robust dual‐anchoring mechanism at the heterointerface. Electronically, the continuous BIEF‐driven charge transfer establishes a powerful Coulombic electrostatic attraction between the electron‐depleted oxyhydroxide surface (δ^+^) and the electron‐accumulated CeO_2_ phase (δ^−^), acting as an “electronic glue” that prevents physical detachment during violent gas evolution. Chemically, the intact M─O─Ce (M = Fe, Co, Ni, Mn) covalent bridging bonds at the interface serve as structural rivets. Since CeO_2_ is electrochemically inert to anodic dissolution, these robust interfacial bonds effectively halt the W‐leaching process at the near‐surface region, enforcing the “self‐limiting” nature of the reconstruction and comprehensively protecting the bulk matrix from over‐leaching and pulverization. This macroscopic self‐limiting behavior is in perfect agreement with our subsequent thermodynamic calculations (detailed in Section 2.4), which reveal that while the initial oxygen vacancy formation is highly thermodynamically favored, any continuous bulk lattice consumption via the lattice oxygen mechanism (LOM) is fundamentally blocked by a prohibitively high kinetic barrier. This conclusion is further supported by the high‐resolution O 1s XPS analysis, which implies a vacancy‐filling mechanism where pre‐existing Ov sites serve as anchors for OH^−^ adsorption. These spectra are also clearly illustrated with the contour plots shown in Figure 5b. High‐resolution O 1s XPS analysis elucidates the dynamic evolution of the catalyst surface (Figure S11). The spectrum reveals a significant decrease in the Ov population, with values falling from 31.98% to 21.71%, alongside a strengthened signal associated with the hydroxide bond (O_OH_). This inverse correlation strongly implies a vacancy‐filling mechanism, wherein pre‐existing Ov sites serve as anchors for OH^−^ adsorption, driving the rapid surface reconstruction into catalytically active M‐OOH species under OER conditions. Further insights into the electronic interplay within this reconstructed surface were provided by examining the valence states of the constituent elements. The high‐resolution XPS spectra of Ce (Figure S12) reveal a marked decrease in the Ce^4^^+^/Ce^3^^+^ ratio from 4.17 (pristine) to 0.99 (post‐OER). This pronounced reductive transition—occurring in a highly anodic oxidation environment—provides compelling evidence that the interfacial Ce species function as a robust dynamic electron sink (buffer). By accepting the delocalized electrons generated during the profound anodic oxidation of the adjacent transition metals (e.g., Ni^2^^+^ to Ni^3^^+^), the Ce domain effectively stabilizes the local electronic density and prevents the oxidative collapse of the reconstructed active layer. Most intriguingly, the deconvoluted spectra of Ni, Co, and Fe reveal an increased proportion of high‐valence M^3+^ species, commonly recognized as active centers for OER. However, the overall binding energies of these metals exhibit a counterintuitive negative shift compared to the pristine state (Figure S13). This can be attributed to the “ligand release” effect caused by W‐leaching, as observed in the in situ Raman analysis. In the pristine lattice, W^6+^ acts as a strong electron‐withdrawing center, creating an electron‐deficient environment around neighboring transition metals. Upon reconstruction, the removal of W^6+^ alleviates this inductive effect. As a result, despite the formation of M^3+^ species, the metal centers reside in a relatively electron‐rich environment modulated by the Ce redox couple. This optimized electronic structure balances the adsorption energies of oxygen intermediates (^*^OH, ^*^O, ^*^OOH), mitigates over‐binding, breaks the scaling relationship, and accelerates the kinetics of ampere‐level water oxidation. In situ Fourier transform infrared (FTIR) spectroscopy was subsequently employed to gain atomistic insights into the OER mechanism and capture the dynamic evolution of reactive intermediates on the catalyst surface. Figure 5c displays the potential‐dependent infrared spectra recorded from open circuit potential (OCP) to 2.15 V vs. RHE.As the applied potential increases, a distinct negative absorption band gradually evolves at approximately 1240 cm^−1^. This feature is unambiguously assigned to the bending vibration (δ) of the ^*^OOH intermediate, serving as a critical spectroscopic signature that the steady‐state catalysis exclusively follows the AEM. The continuous intensification of this negative peak signifies the accumulation of ^*^OOH species on the active sites driven by the anodic bias. Concomitantly, prominent positive bands are observed at ∼1630 and ∼3250 cm^−1^, which are ascribed to the H─O─H bending vibration of water molecules and the O─H stretching vibration of surface hydroxyls (^*^OH) or interfacial water (Figure 5d), respectively [31, 32, 33]. The positive sign of these bands, in contrast to the negative ^*^OOH feature, is indicative of the displacement or consumption of interfacial water molecules and pre‐adsorbed ^*^OH species as they are converted into ^*^OOH intermediates or displaced by the accumulation of catalytic products. The distinct observation of the ^*^OOH intermediate, combined with our macro‐kinetic data, provides direct evidence that the steady‐state OER on this highly polarized reconstructed surface proceeds via the AEM pathway. Importantly, the strong local electric field generated by the BIEF drives this pathway via non‐concerted proton‐electron transfer (non‐CPET) steps, which effectively decouples the pre‐equilibrium deprotonation from the subsequent electron transfer. This unique dynamic is macroscopically corroborated by our pH‐dependent kinetic evaluations (Figure S14). The distinct variation of OER activity across different pH environments on the reversible hydrogen electrode (RHE) scale explicitly confirms that the chemical deprotonation step is effectively decoupled from the electrochemical electron transfer, a classic hallmark of non‐CPET kinetics induced by strong interfacial polarization. Furthermore, the substantial accumulation of ^*^OOH at high potentials suggests that the deprotonation of ^*^OH to form ^*^O (or the subsequent nucleophilic attack to form ^*^OOH) is kinetically favorable, while the subsequent transformation of ^*^OOH highlights its pivotal role in the oxygen evolution cycle.

### 2.4 DFT Calculations

Understanding the atomistic origin of the enhanced catalytic activity motivated the implementation of density functional theory (DFT) calculations. The structural models of CeO_2_, HEW, and the HEW‐CeO_2_ heterostructure are illustrated in Figure 5e. Before delving into the interfacial catalytic mechanisms, the structural geometry and OER thermodynamic trends of the components were rigorously benchmarked against experimental XRD data and various Hubbard U correction settings for Ce 4f states (Table S4 and Figure S15), confirming that the standard GGA functional provides exceptional geometric accuracy and qualitatively consistent energy landscapes for our specific hetero‐system. The prerequisite for the formation of a BIEF is established by the Φ difference between the two components, with HEW exhibiting a lower WF (5.347 eV) compared to CeO_2_ (5.63 eV) [34]. This potential offset drives spontaneous electron migration from the high‐Entropy tungstate to the CeO_2_ domain upon contact, thereby generating a directional BIEF across the heterointerface [35]. The resulting electron redistribution is corroborated by the charge density difference map (Figure 5f), which reveals pronounced electron depletion (cyan regions) on the HEW side and electron accumulation (yellow regions) at the CeO_2_ interface. This spatial charge separation confirms strong interfacial electronic coupling and the directionality of the BIEF toward the semiconductor, effectively modulating the electrophilicity of the metal active sites. To further elucidate the bonding nature and topological continuity of the heterostructure, the electron localization function (ELF) was analyzed (Figure S16). The cross‐sectional ELF map reveals a rigid ionic lattice with highly localized electron regions in the bulk (red, ELF ≈ 0.8–1.0), while the interface displays continuous channels of moderate electron density (green, ELF ≈ 0.5). This “electron‐gas‐like” distribution indicates orbital hybridization between the tungstate and CeO_2_ layers, forming a low‐resistance conduit that facilitates efficient interfacial charge transfer. As shown in Figure 5g, the total density of states (DOS) analysis further confirms enhanced conductivity in the heterostructure, as evidenced by the increased DOS at the Fermi level for HEW‐CeO_2_, attributed to interfacial electronic interactions [36]. To establish the correlation between electronic structure and catalytic performance, the adsorption free energies of oxygen intermediates were calculated for different models (Figure S17). The elementary steps of the OER, involving ^*^OH, ^*^O, and ^*^OOH intermediates, are depicted in Figure 5h. Notably, to theoretically validate the BIEF‐triggered ‘self‐limiting’ reconstruction observed in our experiments, we calculated the oxygen vacancy formation energy (E_f, Ov_) of the W─O bonds. The pure HEW surface exhibits a highly endothermic E_f, Ov_ of 0.72 eV, whereas the strong interfacial charge transfer driven by the BIEF drastically lowers this energy to an exothermic −1.52 eV (Figure S18). This profound thermodynamic shift confirms that the BIEF acts as the precise trigger for the initial structural activation, making the localized sacrificial leaching of W thermodynamically spontaneous. Crucially, while this initial vacancy generation is facile, further calculations reveal that a continuous consumption of lattice oxygen (a sustained LOM process) faces a prohibitively high thermodynamic barrier of 2.33 eV (Figure S19). This enormous energetic penalty acts as an intrinsic ‘thermodynamic brake’, arresting the W‐leaching process once the optimal active layer is formed. Consequently, the steady‐state OER exclusively proceeds via the highly favorable AEM, with the rate‐determining step (^*^OH to ^*^O) requiring a significantly reduced energy barrier of only 1.58 eV on the HEW‐CeO_2_ heterostructure [37]. Collectively, these theoretical results unambiguously demonstrate that the engineered interface not only triggers and limits the phase evolution but also significantly enhances the steady‐state AEM kinetics. Based on these dynamic findings, a comprehensive mechanism—featuring an initial BIEF‐triggered surface reconstruction followed by a steady‐state four‐step proton‐coupled electron transfer (AEM)—is proposed for the HEW‐CeO_2_ system (Figure 5i) [38].

## 3 Conclusion

In summary, this study establishes a transformative interfacial electric field engineering paradigm for designing resilient high‐entropy electrocatalysts targeted at industrial‐scale water oxidation. It is demonstrated that the work function‐induced BIEF at the HEW‐CeO_2_ interface serves as a critical regulator for both electronic structures and dynamic phase transformations. A controlled sacrificial leaching mechanism is uncovered, wherein the BIEF‐driven extraction of W species facilitates the in situ generation of a highly active FeCoNiMn‐OOH layer. Crucially, this reconstructed layer is strongly anchored to the CeO_2_ support via interfacial pinning, ensuring structural integrity even under the intense mechanical stress of gas evolution at ampere‐level current densities. By bypassing conventional linear scaling relationships through the AEM pathway, the heterostructure achieves an outstanding overpotential of 337 mV at 1000 mA cm^−2^ combined with exceptional long‐term chronoamperometric durability. These findings offer a universal strategy for coupling interfacial electric fields with tailored surface chemistry to create next‐generation catalysts for extreme energy conversion environments.

## Author Contributions

**Bing Zhang**: investigation, formal analysis, Writing – review and editing. **Liang Yan**: conceptualization, writing – original draft, writing – review and editing, funding acquisition. **Hao Li**: supervision, project administration, resources. **Yueqi Zhang**: investigation, methodology, data curation. **Jingyi Ye**: data curation, validation, visualization.

## Conflicts of Interest

The authors declare no conflicts of interest.

## Supporting information




**Supporting File**: advs77140‐sup‐0001‐SuppMat.docx.

## Data Availability

The data that support the findings of this study are available from the corresponding author upon reasonable request.

## References

[1] G. Ruan , F. Todman , G. Yogev , et al., “Technologies and Prospects for Decoupled and Membraneless Water Electrolysis,” Nature Reviews Clean Technology 1 (2025): 380–395, 10.1038/s44359-025-00061-1.

[2] A. Odenweller and F. Ueckerdt , “The Green Hydrogen Ambition and Implementation Gap,” Nature Energy 10 (2025): 110–123, 10.1038/s41560-024-01684-7.

[3] B. Liu , H. Zhong , J. Liu , et al., “Modulation of Electrochemical Reactions Through External Stimuli: Applications in Oxygen Evolution Reaction and Beyond,” ACS Nano 19 (2025): 5110–5130, 10.1021/acsnano.5c00099. 39878872

[4] R. Qin , G. Chen , X. Feng , J. Weng , and Y. Han , “Ru/Ir‐Based Electrocatalysts for Oxygen Evolution Reaction in Acidic Conditions: From Mechanisms, Optimizations to Challenges,” Advanced Science 11 (2024): 2309364, 10.1002/advs.202309364. 38501896 PMC11151080

[5] S. Yang , X. Liu , S. Li , et al., “The Mechanism of Water Oxidation Using Transition Metal‐Based Heterogeneous Electrocatalysts,” Chemical Society Reviews 53 (2024): 5593–5625, 10.1039/D3CS01031G. 38646825

[6] B. Wu , S. Gong , Y. Lin , et al., “A Unique Niooh@Feooh Heteroarchitecture for Enhanced Oxygen Evolution in Saline Water,” Advanced Materials 34 (2022): 2108619, 10.1002/adma.202108619. 36055645

[7] S.‐H. Li , M.‐Y. Qi , Z.‐R. Tang , and Y.‐J. Xu , “Nanostructured Metal Phosphides: From Controllable Synthesis to Sustainable Catalysis,” Chemical Society Reviews 50 (2021): 7539–7586, 10.1039/D1CS00323B. 34002737

[8] H. Wang , J. Li , K. Li , et al., “Transition Metal Nitrides for Electrochemical Energy Applications,” Chemical Society Reviews 50 (2021): 1354–1390, 10.1039/D0CS00415D. 33295369

[9] R. Luo , Z. Qian , L. Xing , et al., “Re‐Looking Into the Active Moieties of Metal X‐Ides (X‐ = Phosph‐, Sulf‐, Nitr‐, and Carb‐) Toward Oxygen Evolution Reaction,” Advanced Functional Materials 31 (2021): 2102918, 10.1002/adfm.202102918.

[10] F. Meharban , C. Lin , X. Wu , et al., “Scaling Up Stability: Navigating From Lab Insights to Robust Oxygen Evolution Electrocatalysts for Industrial Water Electrolysis,” Advanced Energy Materials 14 (2024): 2402886, 10.1002/aenm.202402886.

[11] P. A. Kempler , R. H. Coridan , and L. Luo , “Gas Evolution in Water Electrolysis,” Chemical Reviews 124 (2024): 10964–11007, 10.1021/acs.chemrev.4c00211. 39259040

[12] X. Yan , Y. Zhou , and S. Wang , “Nano‐High Entropy Materials in Electrocatalysis,” Advanced Functional Materials 35 (2025): 2413115, 10.1002/adfm.202413115.

[13] Y. Zhang , Y. Yang , B. Lu , et al., “Nickel Tungstate‐Based Electrocatalyst, Photocatalyst, and Photoelectrocatalyst in Water Splitting Applications,” International Journal of Hydrogen Energy 53 (2024): 859–874, https://www.sciencedirect.com/science/article/pii/S0360319923061335.

[14] X. Zhao , M. Liu , Y. Wang , et al., “Designing a Built‐In Electric Field for Efficient Energy Electrocatalysis,” ACS Nano 16 (2022): 19959–19979, 10.1021/acsnano.2c09888. 36519975

[15] S. Hong , H. G. Abbas , K. Jang , et al., “Tuning the C_1_/C_2_ Selectivity of Electrochemical CO_2_ Reduction on Cu–CeO_2_ Nanorods by Oxidation State Control,” Advanced Materials 35 (2023): 2208996, 10.1002/adma.202208996. 36470580

[16] L. Wang , R. Du , Z. Zhao , et al., “Proton‐Conducting, Vacancy‐Rich Hxiroy Nanosheets for the Fabrication of Low‐Ionomer‐Dependent Anode Catalyst Layer in PEM Water Electrolyzer,” Angewandte Chemie International Edition 64 (2025): 202501744, 10.1002/anie.202501744. 40223344

[17] Y. Li , Z. Zhang , Z. Zhang , et al., “Construction of Ni_2_P‐NiFe_2_O_4_ Heterostructured Nanosheets Towards Performance‐Enhanced Water Oxidation Reaction,” Applied Catalysis B: Environmental 339 (2023): 123141, https://www.sciencedirect.com/science/article/pii/S0926337323007841.

[18] F. Luo , R. Xu , S. Ma , et al., “Engineering Oxygen Vacancies of Cobalt Tungstate Nanoparticles Enable Efficient Water Splitting in Alkaline Medium,” Applied Catalysis B: Environmental 259 (2019): 118090, https://www.sciencedirect.com/science/article/pii/S0926337319308379.

[19] L. Qi , Z. Zheng , C. Xing , et al., “1D Nanowire Heterojunction Electrocatalysts of MnCo_2_O_4_/GDY for Efficient Overall Water Splitting,” Advanced Functional Materials 32 (2022): 2107179, 10.1002/adfm.202107179.

[20] Y. Zhao , Y. Ding , W. Li , et al., “Efficient Urea Electrosynthesis From Carbon Dioxide and Nitrate Via Alternating Cu–W Bimetallic C–N Coupling Sites,” Nature Communications 14 (2023): 4491, 10.1038/s41467-023-40273-2. PMC10372083 37495582

[21] R. Huang , Z. Zhai , X. Chen , et al., “Constructing Built‐In Electric Field in NiCo_2_O_4_‐CeO_2_ Heterostructures to Regulate Li_2_O_2_ Formation Routes at High Current Densities,” Small 20 (2024): 2310808, 10.1002/smll.202310808. 38386193

[22] J. Yang , Y. Wang , J. Yang , et al., “Quench‐Induced Surface Engineering Boosts Alkaline Freshwater and Seawater Oxygen Evolution Reaction of Porous NiCo_2_O_4_ Nanowires,” Small 18 (2022): 2106187, https://onlinelibrary.wiley.com/doi/abs/10.1002/smll.202106187. 10.1002/smll.202106187 34862718

[23] W. Luo , H. Tian , Q. Li , et al., “Controllable Electron Distribution Reconstruction of Spinel NiCo_2_O_4_ Boosting Glycerol Oxidation at Elevated Current Density,” Advanced Functional Materials 34 (2024): 2306995, 10.1002/adfm.202306995.

[24] G. Zhao , G. Hai , P. Zhou , et al., “Electrochemical Oxidation of 5‐Hydroxymethylfurfural on CeO_2_‐Modified Co_3_O_4_ With Regulated Intermediate Adsorption and Promoted Charge Transfer,” Advanced Functional Materials 33 (2023): 2213170, 10.1002/adfm.202213170.

[25] K. Wang , S. Wang , K. S. Hui , et al., “Dense Platinum/Nickel Oxide Heterointerfaces With Abundant Oxygen Vacancies Enable Ampere‐Level Current Density Ultrastable Hydrogen Evolution in Alkaline,” Advanced Functional Materials 33 (2023): 2211273, 10.1002/adfm.202211273.

[26] X. Yu , H. Tian , Z. Fu , et al., “Strengthening the Hydrogen Spillover Effect Via the Phase Transformation of W_18_O_49_ for Boosted Hydrogen Oxidation Reaction,” ACS Catalysis 13 (2023): 2834–2846, 10.1021/acscatal.2c04174.

[27] P. Zhou , G. Hai , G. Zhao , et al., “CeO_2_ as an “Electron Pump” to Boost the Performance of Co_4_N in Electrocatalytic Hydrogen Evolution, Oxygen Evolution and Biomass Oxidation Valorization,” Applied Catalysis B: Environmental 325 (2023): 122364, https://www.sciencedirect.com/science/article/pii/S0926337323000073.

[28] J. Liao , Q. Wu , X. Ye , et al., “Visible‐Light‐Induced Electron Density Enrichment of the Active Sites in the Core‐Satellite Structured CuWO_4_@NiO for Fast Hydrogen Generation From Ammonia Borane Methanolysis,” Chemical Engineering Journal 476 (2023): 146599, https://www.sciencedirect.com/science/article/pii/S1385894723053305.

[29] X. Ding , R. Jiang , J. Wu , et al., “Ni_3_N–CeO_2_ Heterostructure Bifunctional Catalysts for Electrochemical Water Splitting,” Advanced Functional Materials 33 (2023): 2306786, 10.1002/adfm.202306786.

[30] J. Zhu , L. Li , and M. Cao , “Le Chatelier's Principle to Stabilize Intrinsic Surface Structure of Oxygen‐Evolving Catalyst for Enabling Ultra‐High Catalytic Stability of Zinc‐Air Battery and Water Splitting,” Nano Energy 122 (2024): 109300, https://www.sciencedirect.com/science/article/pii/S221128552400048X.

[31] S. Li , T. Liu , W. Zhang , et al., “Highly Efficient Anion Exchange Membrane Water Electrolyzers Via Chromium‐Doped Amorphous Electrocatalysts,” Nature Communications 15 (2024): 3416, 10.1038/s41467-024-47736-0. PMC11035637 38649713

[32] X. Luo , H. Zhao , X. Tan , et al., “Fe‐S Dually Modulated Adsorbate Evolution and Lattice Oxygen Compatible Mechanism for Water Oxidation,” Nature Communications 15 (2024): 8293, 10.1038/s41467-024-52682-y. PMC11436974 39333518

[33] Y. Qin , X. Niu , R. Zhao , et al., “Manganese as Electron Reservoir Stabilized RuMnO_x_@RuO_x_ With Enhanced Activity and Robust Durability for Acidic Water Oxidation,” ACS Catalysis 14 (2024): 12970, 10.1021/acscatal.4c01707.

[34] W. J. Zhang , L. Yang , Z. Li , et al., “Regulating Hydrogen/Oxygen Species Adsorption Via Built‐In Electric Field ‐Driven Electron Transfer Behavior at the Heterointerface for Efficient Water Splitting,” Angewandte Chemie International Edition 63 (2024): 202400888, 10.1002/anie.202400888. 38419146

[35] Y. Liu , J. Ding , F. Li , et al., “Modulating Hydrogen Adsorption Via Charge Transfer at the Semiconductor–Metal Heterointerface For Highly Efficient Hydrogen Evolution Catalysis,” Advanced Materials 35 (2023): 2207114, 10.1002/adma.202207114. 36205652

[36] Z. Jiang , S. Song , X. Zheng , et al., “Lattice Strain and Schottky Junction Dual Regulation Boosts Ultrafine Ruthenium Nanoparticles Anchored on a N‐Modified Carbon Catalyst for H_2_ Production,” Journal of the American Chemical Society 144 (2022): 19619–19626, 10.1021/jacs.2c09613. 36223550

[37] S. C. Zhang , C. H. Tan , R. P. Yan , et al., “Constructing Built‐In Electric Field in Heterogeneous Nanowire Arrays for Efficient Overall Water Electrolysis,” Angewandte Chemie International Edition 62 (2023): 202302795, 10.1002/anie.202302795. 37046392

[38] X. Wang , H. Zhong , S. Xi , W. S. V. Lee , and J. Xue , “Understanding of Oxygen Redox in the Oxygen Evolution Reaction,” Advanced Materials 34 (2022): 2107956, 10.1002/adma.202107956. 35853837

