# Supplementary material for: Interfacial Electric Field Engineering of High‐Entropy Heterostructures Triggering Self‐Limiting Reconstruction for Industrial‐Scale Electrocatalysis
Source: Adv Sci (Weinh). 2026 Aug 21;13(62):e77140. doi: 10.1002/advs.77140 (PMC13496283; doi:10.1002/advs.77140)
Supplement: Supplementary file 1 [file ADVS-13-e77140-s001.docx]

**Interfacial Electric Field Engineering of High-Entropy Heterostructures Triggering Self-Limiting Reconstruction for Industrial-Scale Electrocatalysis**

Liang Yan^1^*, Yueqi Zhang^1^, Bing Zhang^2^, jingyi Ye^1^, Hao Li^1^*

^1^ School of Chemistry and Materials Engineering, Huizhou University, Huizhou 516007, China

^2^ School of Intelligent Manufacturing, Huzhou College, Huzhou 313000, China

*Corresponding authors:

E-mail: [yanliang@hzu.edu.cn](mailto:yanliang@hzu.edu.cn); [lihao180@126.com](mailto:lihao180@126.com)

**Experimental Procedures**

***1. Chemical reagents***

Co(NO_3_)_2_·6H_2_O, Fe(NO_3_)_3_·9H_2_O, Ni(NO_3_)_2_·6H_2_O, MnSO_4_·H_2_O, Ce(NO_3_)_3_·6H_2_O, Na_2_WO_4_·2H_2_O, RuO_2_, urea, N-methyl-2-pyrrolidone (NMP), and anhydrous ethanol were all purchased from Shanghai Macklin Biochemical Co., Ltd. and used as received without further purification. Nickel foam (99.9%, thickness: 2 mm, pore size: 110 ppi) was obtained from Suzhou Keshen Metal Materials Co., Ltd. Deionized water (resistivity ≥ 18.2 MΩ·cm) was self-prepared and used for all solution preparations.

***2. Synthesis of catalysts***

Nickel foam pretreatment: Nickel foam (2 cm × 2 cm) was sequentially sonicated in anhydrous ethanol, 3 M HCl, and deionized water for 10 min each, followed by drying in a vacuum oven at 60 °C for subsequent use.

***Synthesis of FeCoNiMnWO_4_-CeO_2_/NF:*** Typically, Co(NO_3_)_2_·6H_2_O (3.0 mmol), Fe(NO_3_)_3_·9H_2_O (3.0 mmol), Ni(NO_3_)_2_·6H_2_O (3.0 mmol), MnSO_4_·H_2_O (3.0 mmol), Ce(NO_3_)_3_·6H_2_O (3.0 mmol), and urea (20.0 mmol) were dissolved in 40 mL of deionized water to form solution A. Separately, Na_2_WO_4_·2H_2_O (15.0 mmol) was dissolved in 40 mL of deionized water to form solution B. The two solutions were mixed thoroughly, and a piece of pretreated nickel foam was immersed in the resulting mixture. The suspension was transferred into a Teflon-lined stainless-steel autoclave and subjected to hydrothermal treatment at 170 °C for 6 h. The obtained solid was collected, dried, and subsequently calcined in air at 500 °C for 2.5 h to yield FeCoNiMnWO_4_-CeO_2_/NF.

***Synthesis of FeCoNiMnWO_4_/NF:*** The synthesis of FeCoNiMnWO_4_/NF followed an identical protocol to that of FeCoNiMnWO_4_-CeO_2_/NF, with the exception that Ce(NO_3_)_3_·6H_2_O was not added to the reaction mixture.

***Synthesis of CoNiWO_4_/NF:*** CoNiWO_4_/NF was prepared similarly, except that only Co(NO_3_)_2_·6H_2_O and Ni(NO_3_)_2_·6H_2_O were used as the metal precursors in Solution A.

***Synthesis of CeO_2_/NF:*** CeO_2_/NF was synthesized following an identical protocol, with the exclusion of Co, Ni, Fe, and Mn salts from the reaction mixture.

***Synthesis of RuO_2_/NF*:** Commercial RuO_2_ powder (5 mg) was ultrasonically dispersed in 1.0 mL of 0.1 wt % Nafion/isopropanol solution for 30 min to form a homogeneous catalyst ink. Subsequently, 200 μL of the ink was drop-cast multiple times onto a 1 × 1 cm^2^ nickel foam electrode and dried under an infrared lamp. The electrode was then stored in a vacuum oven at 60 °C until use.

***3. Physical Characterization***

The morphology and microstructure of the synthesized catalysts were characterized by scanning electron microscopy (SEM, Hitachi SU8220) and transmission electron microscopy (TEM, FEI Talos F200X). Powder X-ray diffraction (XRD) patterns were recorded on a Bruker D8 Advance diffractometer over a 2θ range of 10–80° to analyze the crystallographic structure. X-ray photoelectron spectroscopy (XPS, Thermo ESCALAB 250Xi) with a monochromatic Al Kα source was employed to determine the surface elemental composition and chemical states. *In situ* Raman spectroscopy was conducted using a LabRam HR800 spectrometer with a 532 nm laser to probe molecular structures and chemical bonding under applied potentials. *In situ* Fourier-transform infrared (FTIR) spectroscopy was performed on a Bruker INVENIO S spectrometer equipped with a liquid-nitrogen-cooled mercury cadmium telluride (MCT) detector. The *in situ* cell used 1.0 M KOH as the electrolyte, with Ag/AgCl and Pt sheets (pretreated with Ar for 1 h) serving as the reference and counter electrodes, respectively. IR spectra were collected under different applied potentials. Fe, Co, and Ni K-edge X-ray absorption fine structure (XAFS) measurements were carried out at the BL11B beamline of the Shanghai Synchrotron Radiation Facility (SSRF, Shanghai, China) using Si(111) double-crystal monochromators. Prior to measurement, samples were pressed into 1 cm diameter pellets and sealed with Kapton tape. The extended X-ray absorption fine structure (EXAFS) and X-ray absorption near-edge structure (XANES) spectra were collected in fluorescence mode. No significant variation in the line shape or peak position was observed between repeated XANES scans for each sample. Data processing and fitting were performed using the Athena and Artemis software packages.

***4. Electrochemical Characterization***

All electrochemical measurements were conducted on a CHI760E electrochemical workstation using a standard three-electrode configuration. The self-supported catalyst (1 × 1 cm²) served as the working electrode, with a carbon rod and an Hg/HgO electrode as the counter and reference electrodes, respectively. All tests were performed in 1.0 M KOH at 25 °C. Potentials were converted to the reversible hydrogen electrode (RHE) scale using the equation:

𝐸_RHE_ = 𝐸_Hg/HgO_ + 0.098 + 0.059×pH

OER activity was evaluated by LSV at a scan rate of 5 mV s^-1^ in O_2_-saturated electrolyte with 85 % iR compensation. Electrochemical impedance spectroscopy (EIS) was performed over a frequency range of 0.1 Hz to 100 kHz with an AC amplitude of 5 mV. The electrochemical double-layer capacitance (C_dl_) was determined from cyclic voltammetry (CV) at various scan rates in the non-Faradaic potential region. Catalyst durability was assessed by accelerated durability tests (ADT) and chronoamperometry (CA).

***5. Computational details***

The spin-polarized density functional theories (DFT) were carried out by using the Vienna Ab initio Simulation Package (VASP).[1] The Perdew-Burke-Ernzerhof generalized-gradient approximation functional was used to describe the interaction between electrons.[2] Furthermore, methodological benchmarking regarding the lattice parameters and the Hubbard U correction for Ce 4f states was systematically conducted to justify the chosen parameters (see Section 6 for details). The energy cutoff was set to 400 eV. The Monkhorst k-points grids was set to 2$\times2\times$1 during the calculations. A vacuum of 20 $Å$ was adopted to prevent the interaction between two adjacent layers. The energy and force convergence was set to 10^-5^ eV and 0.02 eV/$Å$. Bottom layers of the structures are fixed during the calculations.

The reaction Gibbs free energy (ΔG) is defined as ΔG = ΔE + ΔZPE - TΔS, ΔE is the reaction energy, ΔZPE is zero-point energies, T is the temperature (298.15 K), ΔS is the difference in entropy from vibrational frequency calculations. The entropy of gas phase is obtained from the NIST database with standard condition (http://cccbdb.nist.gov/).

***6. Methodological Benchmarking for DFT Calculations***

To validate the reliability of the computational parameters, we performed systemic benchmarks on the bulk lattice parameters and surface OER energetics of CeO_2_ using both standard GGA and GGA+U methods. As compiled in Table S4, the lattice parameter obtained by standard GGA (a = 5.422 Å) aligns exceptionally well with the experimental value from the standard PDF card (a = 5.411 Å, error ~0.2%), whereas the inclusion of U slightly overestimates the parameters (~5.467 Å). Furthermore, the potential-energy diagrams for OER on CeO_2_(111) with various U_eff_ values (4 ~ 6 eV) demonstrate that while absolute adsorption energies shift slightly, the rate-determining step and the overall endothermic catalytic profile remain qualitatively unchanged (Figure S11). These results justify the utilization of standard GGA for the large-scale high-entropy heterostructure to balance computational efficiency and geometric precision.

**Figure S1.** XRD patterns of (a) HEW and (b) CoNiWO_4_. SEM images of (c) HEW and (d) CoNiWO_4_.

**Figure S2.** XPS survey spectrum of HEW-CeO_2_.

**Figure S3.** (a) High-resolution XPS spectra of O 1s for as-prepared HEW-CeO_2_ and HEW. (b) EPR spectra of HEW-CeO_2_ and HEW.

**Figure S4.** Calculated work function of (a) HEW and (b) CeO_2_.

**Figure S5.** (a) KPFM potential images and (b) the corresponding surface potential profiles of the HEW–CeO_2_ heterostructure.

**Figure S6.** LSV curves of HEW+CeO_2_ compared with those of other catalysts.

**Figure S7.** CV curves of (a) HEW-CeO_2_, (b) HEW, (c) CoNiWO_4_, and (d) RuO_2_ at different scan rates.

**Figure S8.** HEW-CeO_2_ after OER test. (a) SEM image, (b) low magnification TEM image, and (c) high magnification TEM image.

**Figure S9.** Relative contents of Fe^2+^/Fe^3+^, Co^2+^/Co^3+^, and Mn^2+^/Mn^3+^ in HEW-CeO_2_/NF before and after OER.

**Figure S10.** High-resolution XPS spectra of Ni 2p for HEW-CeO_2_/NF before and after the OER test.

**Figure S11.** High-resolution XPS spectra of O 1s for HEW-CeO_2_/NF before and after the OER test.

**Figure S12.** Comparison of Ce 3d XPS spectra before and after OER test of HEW-CeO_2_/NF.

**Figure S13.** XPS spectra of (a) Fe 2p and (b) Co 2p for HEW-CeO_2_/NF before and after the OER test.

**Figure S14.** pH dependence of OER activity for the HEW-CeO₂ heterostructure.

**Figure S15.** Comparison of the OER free‑energy diagram on the CeO_2_(111) surface under different Hubbard U settings. The curves correspond to GGA (no U, raw data) and GGA+U with U_eff_ = 4, 5, and 6 eV, illustrating how U affects the adsorption energies of key intermediates (*OH, *O, *OOH) while preserving the overall thermodynamic trend.

**Figure S16.** The ELF graph of HEW-CeO_2_.

**Figure S17.** The adsorption configuration of oxygen-containing intermediates on (a) HEW and (b) CeO_2_ for OER in alkaline medium.

**Figure S18.** Atomic models and the calculated oxygen vacancy formation energies (E_f, Ov_) for the pure HEW(111) surface and the HEW-CeO_2_ heterointerface. The green circles highlight the exact positions of the extracted lattice oxygen atoms adjacent to the W sites.

**Figure S19.** Free‑energy diagrams comparing the AEM and LOM pathways on the HEW‑CeO_2_ heterostructure.

**Table S1.** Deconvoluted peaks of O 1s and calculated oxygen vacancy concentration for various samples.

| Samples | O_L_ | O_v_ | O_abs_ | O_v_/O_L_ ratio |
| --- | --- | --- | --- | --- |
| HEW-CeO_2_ | 26.67% | 31.98% | 41.35% | 1.20 |
| HEW | 42.84% | 31.78% | 25.38% | 0.74 |

**Table S2.** XPS fitting parameters for Fe 2p, Co 2p, Ni 2p, Mn 2p, W4f, Ce3d as well as O1s for HEW-CeO_2_, HEW and CeO_2_.

| Catalysts | Peak | Position / eV | Peak | Position / eV |
| --- | --- | --- | --- | --- |
| HEW-CeO_2_ | Fe^2+^2p_3/2_ | 711.63 | Fe^2+^2p_1/2_ | 724.43 |
|  | Fe^3+^2p_3/2_ | 715.04 | Fe^3+^2p_1/2_ | 728.84 |
|  | Co^3+^2p_3/2_ | 780.13 | Co^3+^2p_1/2_ | 795.86 |
|  | Co^2+^2p_3/2_ | 782.90 | Co^2+^2p_1/2_ | 798.69 |
|  | Ni^2+^2p_3/2_ | 855.46 | Ni^2+^2p_1/2_ | 872.69 |
|  | Ni^3+^2p_3/2_ | 857.28 | Ni^3+^2p_1/2_ | 875.69 |
|  | Mn^2+^2p_3/2_ | 642.43 | Mn^2+^2p_1/2_ | 653.55 |
|  | Mn^3+^2p_3/2_ | 645.83 | Mn^3+^2p_1/2_ | 657.63 |
|  | W^6+^4f_7/2_ | 35.50 | W^6+^4f_5/2_ | 37.69 |
|  | Ce^4+^3d_5/2_ | 882.81 | Ce^3+^3d_5/2_ | 885.86 |
|  | Ce^4+^3d_5/2_ | 889.51 | Ce^4+^3d_5/2_ | 898.70 |
|  | Ce^4+^3d_3/2_ | 901.58 | Ce^3+^3d_3/2_ | 904.66 |
|  | Ce^4+^3d_3/2_ | 908.10 | Ce^4+^3d_3/2_ | 917.08 |
|  | O_L_ 1s | 529.03 | O_v_ 1s | 529.82 |
|  | O_a_ 1s | 531.27 |  |  |
| HEW | Fe^2+^2p_3/2_ | 711.37 | Fe^2+^2p_1/2_ | 724.17 |
|  | Fe^3+^2p_3/2_ | 714.46 | Fe^3+^2p_1/2_ | 727.74 |
|  | Co^3+^2p_3/2_ | 780.92 | Co^3+^2p_1/2_ | 796.52 |
|  | Co^2+^2p_3/2_ | 785.07 | Co^2+^2p_1/2_ | 800.86 |
|  | Ni^2+^2p_3/2_ | 855.53 | Ni^2+^2p_1/2_ | 873.20 |
|  | Ni^3+^2p_3/2_ | 857.35 | Ni^3+^2p_1/2_ | 875.76 |
|  | Mn^2+^2p_3/2_ | 642.39 | Mn^2+^2p_1/2_ | 653.80 |
|  | Mn^3+^2p_3/2_ | 645.82 | Mn^3+^2p_1/2_ | 657.62 |
|  | W^6+^4f_7/2_ | 35.42 | W^6+^4f_5/2_ | 37.61 |
|  | O_L_ 1s | 529.38 | O_v_ 1s | 530.38 |
|  | O_a_ 1s | 531.71 |  |  |
| CeO_2_ | Ce^4+^3d_5/2_ | 882.91 | Ce^3+^3d_5/2_ | 885.98 |
|  | Ce^4+^3d_5/2_ | 889.33 | Ce^4+^3d_5/2_ | 898.68 |
|  | Ce^4+^3d_3/2_ | 901.36 | Ce^3+^3d_3/2_ | 904.39 |
|  | Ce^4+^3d_3/2_ | 908.23 | Ce^4+^3d_3/2_ | 917.12 |

**Table S3.** Comparison of electrocatalytic performance of HEW-CeO_2_/NF for OER in 1.0 M KOH with other reported self-supported catalysts.

| Catalysts | Substrate | η_500_  (mV) | Tafel slope  (mV/dec) | Reference |
| --- | --- | --- | --- | --- |
| **HEW-CeO_2_**/NF | **Nickel foam** | **303** | **23.2** | **This work** |
| Ni_3_S_4_/NiFe-LDH/IF | Iron foam | 297 | 30.8 | [3] |
| Mn-doped Ni_2_P/Fe_2_P/NF | Nickel foam | 304 | 53.88 | [4] |
| Co_4_S_3_/Fe_3_S_4_/MoS_2_/IF | Iron foam | 305 | 79 | [5] |
| Ru-Ni_2_P/Fe_2_P | Nickel foam | 308 | 71.02 | [6] |
| D-EHEA-Zr0.25 | / | 309 | 44.9 | [7] |
| NiFeLa LDH/NF | Nickel foam | 309 | 42 | [8] |
| NiFe LDH-CeW/NFF | Nickel-Iron foam | 309 | 64.4 | [9] |
| NiFe-LDH/NF-S-3h | Nickel foam | 309 | 73.5 | [10] |
| S-FeCo1:1/NIF | Nickel-Iron foam | 310 | 65.01 | [11] |
| M(OH)_x_/Fe_3_O_4_/IF | Iron foam | 311 | 57 | [12] |
| FeNiZn/FeNi_3_@NiFe/NF | Nickel foam | 315 | 45.7 | [13] |
| NiFeCo-0.6/NF | Nickel foam | 318 | 38 | [14] |
| NiFe-LDH/NFF | Nickel-Iron foam | 319 | 59.4 | [15] |
| Ni_2_P@FeP@Co_2_P/NF | Nickel foam | 320 | / | [16] |
| TS-NiFe LDH/NF | Nickel foam | 321 | 37.6 | [17] |
| CoWO_4_/Ni_x_Fe_y_S/NIF | Nickel-Iron foam | 322.8 | 45.2 | [18] |
| NF@FS-6T | Nickel foam | 328 | 56.9 | [19] |
| NiFe MOF@NiSe_x_/NF | Nickel foam | 329 | 38.3 | [20] |
| Ni_3_S_2_@NFP | Nickel foam | 329 | 41.8 | [21] |
| Ru-Co_2_P/CoP/CF | Cobalt foam | 330 | 21.15 | [22] |
| NiCoFe-B/NF | Nickel foam | 340 | 30 | [23] |
| CoP/NiO/NF | Nickel foam | 343 | 43.6 | [24] |
| SS-AC-SR | Foam stainless | 347 | 43 | [25] |
| Ir_SA_-NiFe LDH/NiMo | Nickel foam | 350 | 24 | [26] |
| Mo-CoP/NC | Nickel foam | 352 | 46.3 | [27] |
| SSEAE/800 | stainless steel | 355 | 31.4 | [28] |
| Fe-Mo-S/Ni_3_S@NF | Nickel foam | 367 | 98 | [29] |
| NiFe(S)/NM | Nickel mesh | 370 | 41.4 | [30] |
| Fe-(CeS_2_/Ni_3_S_2_)/NF | Nickel foam | 372 | 51.53 | [31] |
| NiS_2_-Ni_2_P/CC | Carbon cloth | 395 | 33.8 | [32] |
| Ag/NiCo LDH-30 | Nickel foam | 397 | 34 | [33] |
| Ni_x_Co_1−x_(PO_3_)_2_/MoO_x_ | Nickel foam | 419 | 67.6 | [34] |
| a-CoNiS/NF | Nickel foam | 434 | 20.18 | [35] |
| CuS–Ni_3_S_2_/CuNi/NF | Nickel foam | 444 | 142 | [36] |

**Table S4.** Comparison of the calculated lattice parameters (a, b, c, α, β, γ) of CeO_2_ using standard GGA (no U) and GGA+U (U_eff_ = 4 ~ 6 eV), benchmarked against the experimental PDF card.

|  | a (Å) | b (Å) | c (Å) | α (°) | β (°) | γ (°) |
| --- | --- | --- | --- | --- | --- | --- |
| Pdf card | 5.41134 | 5.41134 | 5.41134 | 90 | 90 | 90 |
| no U | 5.42233 | 5.42233 | 5.42233 | 90 | 90 | 90 |
| U_eff_ = 4 | 5.46936 | 5.46936 | 5.46936 | 90 | 90 | 90 |
| U_eff_ = 5 | 5.46745 | 5.46745 | 5.46745 | 90 | 90 | 90 |
| U_eff_ = 6 | 5.46745 | 5.46745 | 5.46745 | 90 | 90 | 90 |

**References**

1. G. Kresse, J. Furthmüller, "Efficiency of ab-initio total energy calculations for metals and semiconductors using a plane-wave basis set," *Computation Materials Science* 6 (1996): 15, <https://www.sciencedirect.com/science/article/pii/0927025696000080>.

2. G. Kresse, J. Furthmüller, "Efficient iterative schemes for ab initio total-energy calculations using a plane-wave basis set," *Phys. Rev. B* 54 (1996): 11169, <https://link.aps.org/doi/10.1103/PhysRevB.54.11169>.

3. Y. Liu, J. Guo, X. Liu, et al., "Corrosion-Driven Ni_3_S_4_ Gradient in NiFe-LDH Enables Durable Industrial-Scale Water Electrolysis," *Angewandte Chemie International Edition* 64 (2025): e202516894, <https://doi.org/10.1002/anie.202516894>.

4. Y. Luo, P. Wang, G. Zhang, et al., "Mn-doped nickel–iron phosphide heterointerface nanoflowers for efficient alkaline freshwater/seawater splitting at high current densities," *Chemical Engineering Journal* 454 (2023): 140061, <https://www.sciencedirect.com/science/article/pii/S1385894722055413>.

5. S. Jin, X. Liu, J. Cao, et al., "FeCo-MOF derived Co_4_S_3_/Fe_3_S_4_/MoS_2_ nanosheet arrays on iron foam for overall water splitting in alkaline water /seawater at large-current density," *International Journal of Hydrogen Energy* 61 (2024): 329, <https://www.sciencedirect.com/science/article/pii/S0360319924007420>.

6. X. Li, T. Wu, N. Li, et al., "Vertically Staggered Porous Ni_2_P/Fe_2_P Nanosheets with Trace Ru Doping as Bifunctional Electrocatalyst for Alkaline Seawater Splitting," *Advanced Functional Materials* 34 (2024): 2400734, <https://doi.org/10.1002/adfm.202400734>.

7. Q. Zhang, Q. Guo, Y. Zhang, et al., "Architecting Gradient Hierarchically Porous Catalyst via Negative Mixing Enthalpy High-Entropy Alloy for Durable Water Splitting at Ampere-Level Current Density," *Advanced Functional Materials* 35 (2025): 2414446, <https://doi.org/10.1002/adfm.202414446>.

8. C. Dang Van, S. Kim, M. Kim, M. H. Lee, "Effect of Rare-Earth Element Doping on NiFe-Layered Double Hydroxides for Water Oxidation at Ultrahigh Current Densities," *ACS Sustainable Chem. Eng.* 11 (2023): 1333, <https://doi.org/10.1021/acssuschemeng.2c05060>.

9. M. Li, H.-J. Niu, Y. Li, et al., "Synergetic regulation of CeO_2_ modification and (W_2_O_7_)^2-^ intercalation on NiFe-LDH for high-performance large-current seawater electrooxidation," *Appl. Catal. B: Environ.* 330 (2023): 122612, <https://www.sciencedirect.com/science/article/pii/S0926337323002552>.

10. Z. Wan, Z. Ma, H. Yuan, K. Liu, X. Wang, "Sulfur Engineering on NiFe Layered Double Hydroxide at Ambient Temperature for High Current Density Oxygen Evolution Reaction," *ACS Appl. Energy Mater.* 5 (2022): 4603, <https://doi.org/10.1021/acsaem.2c00018>.

11. Y. Fan, J. Zhang, J. Han, et al., "In situ self-reconstructed hierarchical bimetallic oxyhydroxide nanosheets of metallic sulfides for high-efficiency electrochemical water splitting," *Mater. Horiz.* 11 (2024): 1797, <http://dx.doi.org/10.1039/D3MH02090H>.

12. X. Wang, L. Wang, Y. Liu, et al., "Boosting the oxygen evolution reaction via the reconstruction of an M(OH)_x_/Fe_3_O_4_ catalyst," *Inorg. Chem. Front.* 11 (2024): 6333, <http://dx.doi.org/10.1039/D4QI01574F>.

13. Q. Zhou, C. Xu, J. Hou, et al., "Duplex Interpenetrating-Phase FeNiZn and FeNi_3_ Heterostructure with Low-Gibbs Free Energy Interface Coupling for Highly Efficient Overall Water Splitting," *Nano-Micro Lett.* 15 (2023): 95, <https://doi.org/10.1007/s40820-023-01066-w>.

14. Y. Wu, Y. Li, Z. Xie, et al., "Tracking the structural evolution and activity origin of Co-doped NiFe layered double hydroxide for enhanced oxygen evolution reaction," *Chemical Engineering Journal* 488 (2024): 151086, <https://www.sciencedirect.com/science/article/pii/S1385894724025737>.

15. S. Song, Y. Wang, P. Tian, J. Zang, "Activating lattice oxygen in local amorphous S-modified NiFe-LDH ultrathin nanosheets toward superior alkaline/natural seawater oxygen evolution," *Journal of Colloid and Interface Science* 677 (2025): 853, <https://www.sciencedirect.com/science/article/pii/S0021979724018137>.

16. W. Ma, Y. Zhang, L. Hu, X. Lv, J. Dang, "Dynamic Restructuring of Asymmetric Built-in Electric Field Catalysts Facilitates the Efficient Water Splitting," *Advanced Functional Materials* 35 (2025): 2422734, <https://doi.org/10.1002/adfm.202422734>.

17. H. Wang, Z. Li, S. Hong, et al., "Tungstate Intercalated NiFe Layered Double Hydroxide Enables Long-Term Alkaline Seawater Oxidation," *Small* 20 (2024): 2311431, <https://doi.org/10.1002/smll.202311431>.

18. Q. Qian, C. Chen, X. Zheng, et al., "Hierarchical CoWO_4_/NixFeyS microspheres bearing crystalline-amorphous interface as a multifunctional platform for outperformed water splitting and sensitive hydrazine sensing," *Journal of Colloid and Interface Science* 664 (2024): 756, <https://www.sciencedirect.com/science/article/pii/S0021979724005356>.

19. W. Zhang, X. He, P. Pan, et al., "Strong Magnetic Field-Enhanced Corrosion Engineering Unlocks Integrated Amorphous/Crystalline Bifunctional Electrodes for Stable High-Current Water Electrolysis," *Advanced Functional Materials* 36 (2026): e14469, <https://doi.org/10.1002/adfm.202514469>.

20. W. Hu, Q. Yan, S. Ma, et al., "Surface-selenization formed NiFe MOF@NiSe_x_ heterogeneous arrays for enhanced oxygen evolution and methanol electrooxidation," *Journal of Electroanalytical Chemistry* 975 (2024): 118789, <https://www.sciencedirect.com/science/article/pii/S1572665724007677>.

21. X. Chen, K. Xu, J. Li, et al., "Ni_3_S_2_@NiFeP_x_ electrode with dual-anion-modulated layer for efficient and stable oxygen evolution," *Chinese Chemical Letters* 34 (2023): 108713, <https://www.sciencedirect.com/science/article/pii/S100184172300548X>.

22. J. Xiao, T. Huang, J. Jiang, et al., "Engineering Ru-Complementary Catalytic Centers on Co_2_P/CoP Heterojunction for Industrial Alkaline Water Electrolysis," *Advanced Functional Materials* 35 (2025): e07040, <https://doi.org/10.1002/adfm.202507040>.

23. X. She, C. Feng, D. Liu, et al., "A stable alkaline anion exchange membrane water electrolyzer based on a self-healing anode," *International Journal of Hydrogen Energy* 59 (2024): 1297, <https://www.sciencedirect.com/science/article/pii/S0360319923065904>.

24. Q. Liao, T. You, X. Liu, et al., "Coupled plasma etching and electrodeposition of CoP/NiO nanosheets with surface reconstruction for water-splitting," *J. Mater. Chem. A* 12 (2024): 9830, <http://dx.doi.org/10.1039/D4TA00277F>.

25. C. Hou, L. Xue, J. Li, et al., "Unlocking the potential of lattice oxygen evolution in stainless steel to achieve efficient OER catalytic performance," *Acta Materialia* 277 (2024): 120176, <https://www.sciencedirect.com/science/article/pii/S1359645424005275>.

26. Y. Wu, M. Chen, H. Sun, et al., "Coupling Ir single atom with NiFe LDH/NiMo heterointerface toward efficient and durable water splitting at large current density," *Applied Catalysis B: Environment and Energy* 360 (2025): 124548, <https://www.sciencedirect.com/science/article/pii/S0926337324008622>.

27. W. An, Z. Liu, T. Shen, et al., "Regulating d–p Hybridization in Mo-Doped CoP Nanoflowers with Superwetting Surfaces for Water Splitting at 3000 mA cm^–2^," *ACS Catalysis* 15 (2025): 10358, <https://doi.org/10.1021/acscatal.4c07485>.

28. L. Xue, B. Wang, J. Hu, et al., "Dynamic Self-Optimizing Reconstruction of Stainless Steel Fe-Ni Dual Sites Accelerates Catalytic Intermediate Coupling for High-Efficiency Oxygen Evolution Reaction at Industrial Current Density," *Advanced Functional Materials* 36 (2026): e16748, <https://doi.org/10.1002/adfm.202516748>.

29. Y. Zhang, H. Guo, X. Li, et al., "A 3D multi-interface structure of coral-like Fe-Mo-S/Ni_3_S_2_@NF using for high-efficiency and stable overall water splitting," *Chemical Engineering Journal* 404 (2021): 126483, <https://www.sciencedirect.com/science/article/pii/S1385894720326115>.

30. Q. Li, W. Luo, X. Cui, J. Shi, "Rapid and In Situ Active Sites Regeneration for OER Activity Recovery and Greatly Prolonged Water-Splitting Performance," *Angewandte Chemie International Edition* 64 (2025): e202500303, <https://doi.org/10.1002/anie.202500303>.

31. X. Zhang, X. Wang, Q. Yan, et al., "Defects engineering and interface regulation on nickel-rich sulphides promoting water/urea/ethanol electrooxidation," *Chemical Engineering Journal* 486 (2024): 150397, <https://www.sciencedirect.com/science/article/pii/S1385894724018849>.

32. Y. Wang, R. Yin, L. Yuan, et al., "Uniform Sub-5 nm Crystalline Nickel-Based Heterojunctions for Overall Water Splitting Electrocatalysis," *ACS Energy Letters* 10 (2025): 837, <https://doi.org/10.1021/acsenergylett.4c03097>.

33. Y. Sun, G. Chen, F. El bachraoui, et al., "Ag on NiCo Layered Double Hydroxide as Oxygen Evolution Electrocatalyst for Anion Exchange Membrane Water Electrolyzer Under Large Current Densities," *Small Methods* 9 (2025): 2500103, <https://doi.org/10.1002/smtd.202500103>.

34. P. Wang, P. Wang, T. Wu, X. Sun, Y. Zhang, "Bimetal Metaphosphate/Molybdenum Oxide Heterostructure Nanowires for Boosting Overall Freshwater/Seawater Splitting at High Current Densities," *Advanced Science* 11 (2024): 2407892, <https://doi.org/10.1002/advs.202407892>.

35. X. Wu, S. Zhao, L. Yin, et al., "Amorphous porous sulfides nanosheets with hydrophilic/aerophobic surface for high-current-density water splitting," *Chinese Chemical Letters* 34 (2023): 108016, <https://www.sciencedirect.com/science/article/pii/S1001841722010257>.

36. N. Zhang, Y. Gao, Y. Mei, et al., "CuS–Ni_3_S_2_ grown in situ from three-dimensional porous bimetallic foam for efficient oxygen evolution," *Inorg. Chem. Front.* 6 (2019): 293, <http://dx.doi.org/10.1039/C8QI01148F>.
